# Supplementary material for: Maintaining protein stability of ∆Np63 via USP28 is required by squamous cancer cells
Source: EMBO Mol Med. 2020 Mar 4;12(4):e11101. doi: 10.15252/emmm.201911101 (PMC7136964; doi:10.15252/emmm.201911101)
Supplement: Supplementary file 1 — Appendix [file EMMM-12-e11101-s001.pdf]

Maintaining protein stability of ∆Np63 via USP28 is required by squamous cancer cells

Cristian Prieto-Garcia[1,2], Oliver Hartmann[1,2], Michaela Reissland[1,2], Fabian Braun[1,2], Thomas Fischer[3], Susanne Walz[4], Christina Schülein-Völk[5], Ursula Eilers[5] , Carsten P. Ade[2,13], Marco A. Calzado [6,7,8,], Amir Orian[9], Hans M. Maric[10], Christian Münch[11], Mathias Rosenfeldt[2,12], Martin Eilers [2,13] & Markus E. Diefenbacher[1,2*]

Appendix

Appendix Supplementary Figures (page 2 - 4)

- Appendix Figure S1: USP28 stabilizes ΔNp63 independently of FBXW7. (page 2)
- Appendix Figure S2: USP28 modulates ΔNp63 proteasomal degradation in FBXW7 deficient cells. (page 3)
- Appendix Figure S3: ΔNp63-driven SCC cells of various tissues are vulnerable to USP28 depletion. (page 4)

Appendix supplementary gene-sets used for RNA-seq (page 5 - 21).

- Appendix Table S1 (page 5-20): Genes up and down regulated in shUSP28 /shDNp63 vs shNTC.
- Appendix Table S2 (page 21): Data-set Squamous cancer markers.

Appendix Table S3 (page 22- 31): Exact P-values and statistical tests.

- Fig1 (page 22)
- Fig2 (page 23)
- Fig3 (page 23-24)
- Fig4 (page 24-26)
- Fig5 (page 26)
- Fig6 (page 27)
- Fig8 (page 27)
- Appendix Fig S1 (page28)
- Appendix Fig S2 (page 28)
- Fig. EV2 (page 29-30)
- Fig. EV3 (page 30)
- Appendix Fig S3 (30-31)

Appendix Table S4 (page 32): Antibody dilution list.

Appendix Table S5 (page 33-45): Consumables.

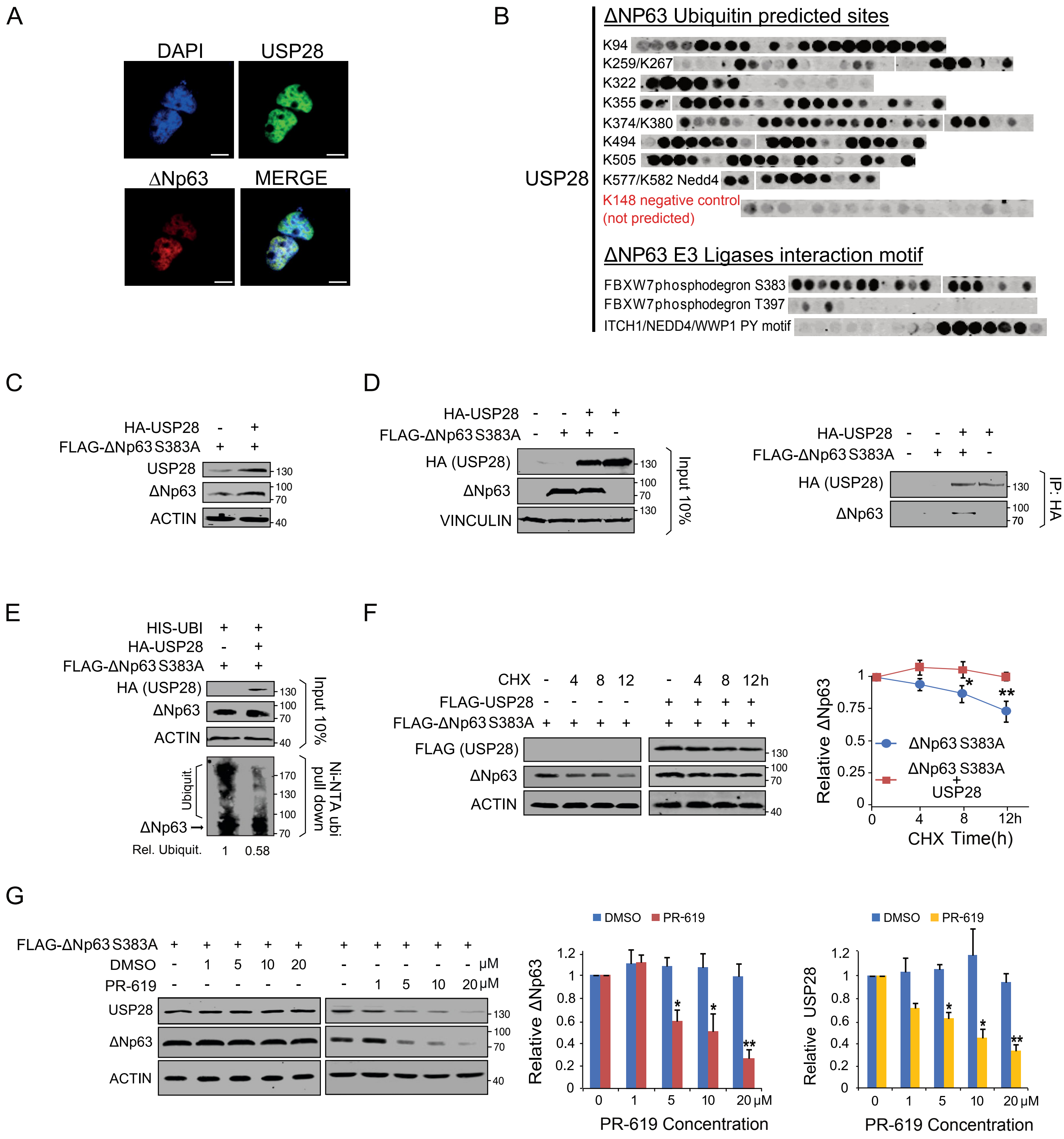

Appendix Figure S1: USP28 stabilizes ΔNp63 independently of FBXW7.

A) Immunofluorescence staining against overexpressed HA-USP28 and FLAG-ΔNp63 in HEK293 cells; Scale bar = 5μm

B) SPOT array immunoblot of endogenous USP28 bound to ΔNP63 peptides. USP28 bound to lysine containing domains within ΔNP63 with a high propability of ubiquitylation (numbers indicate individual lysine residues and see Ubisite and ubpred online tools) and known E3 ligases interaction motifs;

C) Representative Western blot of ΔNp63, USP28 and ACTIN in FLAG-ΔNp63S383A or FLAG-ΔNp63S383A + HA-USP28 HEK293-T transfected cells. ACTIN as a control;

D) Representative co-immunoprecipitation of exogenous HA-USP28 and FLAG-ΔNp63S383A in HEK293 cells. VINCULIN served as loading control. HA-USP28 was precipitated and blotted against FLAG-ΔNp63S383A or HAUSP28 (n=3).

E) Ni-NTA His-ubiquitin pulldown of exogenous FLAG-ΔNp63S383A in control transfected or HA-USP28 overexpressing HEK293 cells, followed by immunoblot against ΔNp63S383A protein (Relative Ubiquitination calculated using ACTIN for normalization. Representative experiment of n=3 ;

F) Immunoblot of HEK293 cells transiently co-transfected with FLAG-ΔNp63S383A together with FLAG-USP28, followed by CHX chase for indicated timepoints. ACTIN served as loading control. Quantification of relative protein abundance of three individual experiments;

G) Immunoblot of HEK293 cells transiently co-transfected with FLAG-ΔNp63S383A. 24 hours later cells were treated with either DMSO or the pan-DUB inhibitor PR619 for 24 hours for indicated concentrations., followed by immunoblot against endogenous USP28 and FLAG-ΔNp63S383A. ACTIN served as loading control. Quantification of relative protein abundance of three individual experiments.

Data information: All quantitative data are represented as mean ± SD; *p < 0.05, **p < 0.01. Two tailed t-test.See also Appendix Table S3.

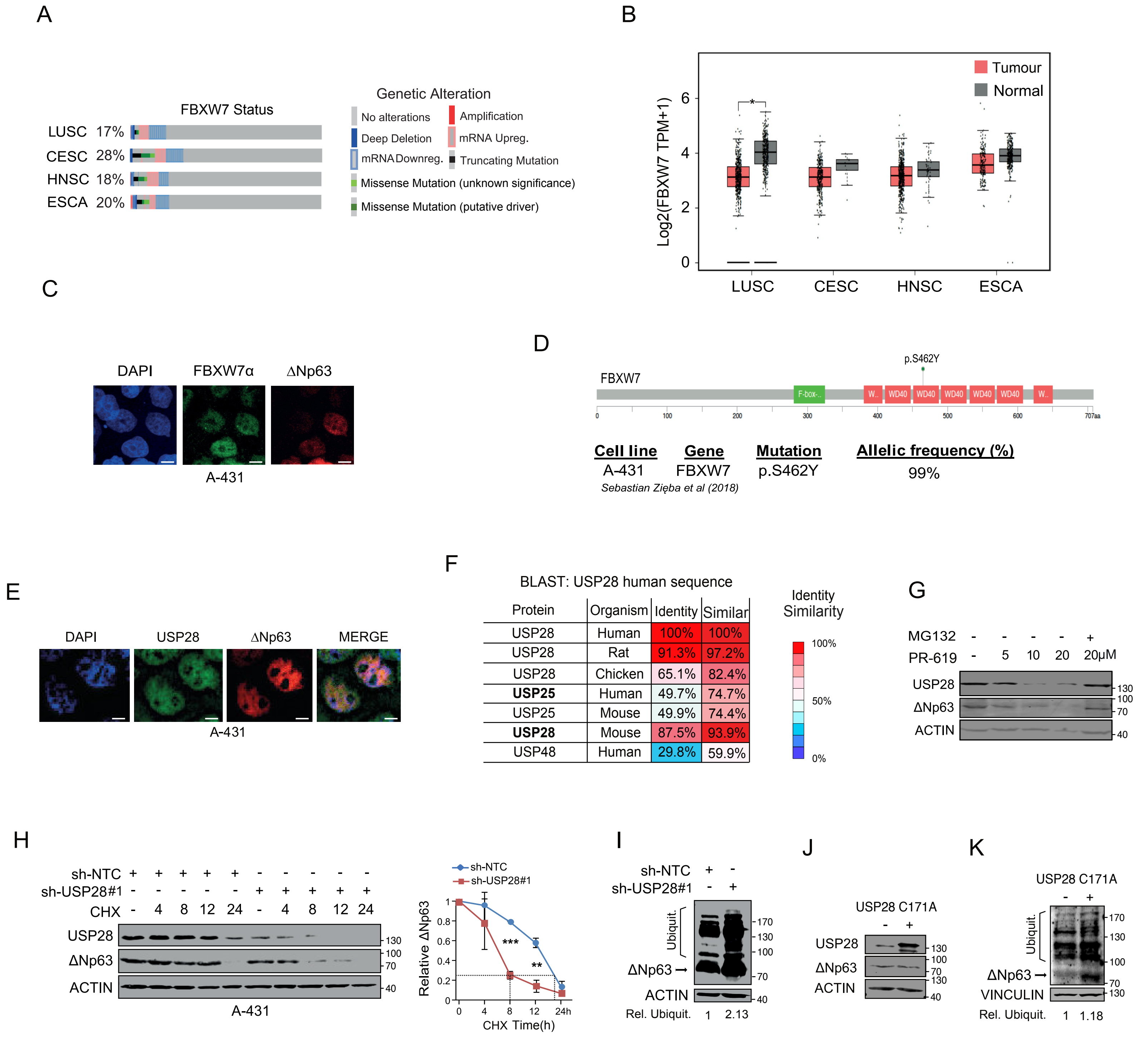

Appendix Figure S2: USP28 modulates ΔNp63 proteasomal degradation in FBXW7 deficient cells.

A) Analysis of occurring genetic alterations of FBXW7 in lung-, cervical-, head and neck- and oesophageal squamous tumours (Cbioportal).
B) Relative mRNA expression data of FBXW7 in human Lung (n=486), Cervix (n=306), Oesophagus (n=182) and HNSC (n=519) SCC tumours and normal non-transformed tissue (nLung=338 nCervix=13, nOesophagus=286 and nHNSC=44).Gepia software. In the box plot, the centre line reflects the median, the cross represents the mean and the upper and lower box limits indicates the first and third quartile. Whiskers extend 1.5x the IQR.
C) Immunofluorescence staining against endogenous FBXW7 and ΔNp63 in the human SCC cell line A-431 (n=3). Scale bar = 5μm
D) Mutational analyisis of FBXW7 in A-431 (Cosmic sanger);
E) Immunofluorescence staining against endogenous USP28 and ΔNp63 in A431.
F) Identity and similarity of USP28 and USP25 in different organisms (European Bioinformatics Institute; EMBL-EBI).
G) Treatment of A-431 cells with the pan-DUB inhibitor PR-619 for 24 hours at indicated time-points, followed by MG132 freatment for 6 hours at the highest PR-619 concentration, followed by Immunoblot against endogenous USP28 and ΔNp63. ACTIN served as loading control. Representative western blot (n=3).
H) CHX chase for indicated timepoints, followed by Immunoblot against endogenous USP28 and ΔNp63, in A-431 cells stably transduced with constitutive shRNA-non-targeting control (NTC) or against USP28. ACTIN served as loading control. Quantification of protein stability of ΔNp63 in three individual experiments.
I) TUBE ubiquitin pulldown against endogenously ubiquitylated ΔNp63 in constitutive shRNA-NTC or USP28 knock down A-431 cells, followed by immunoblot against ΔNp63. ACTIN served as loading control to calculate relative ubiquitination. (n=3).
J) Immunoblot against USP28 and ΔNp63 in control and USP28 C171A A-431 transfected cells. ACTIN served as loading control. n=3.
K) TUBE ubiquitin pulldown against endogenously ubiquitylated ΔNp63 in control or USP28 C171A transfected A-431 cells, followed by immunoblot against ΔNp63. VINCULIN served as loading control to calculate relative ubiquitination. (n=3).

Data information: All quantitative data are represented as mean ± SD; *p < 0.05, **p < 0.01; *** p< 0.001. Two tailed t-test. See also Appendix Table S3.

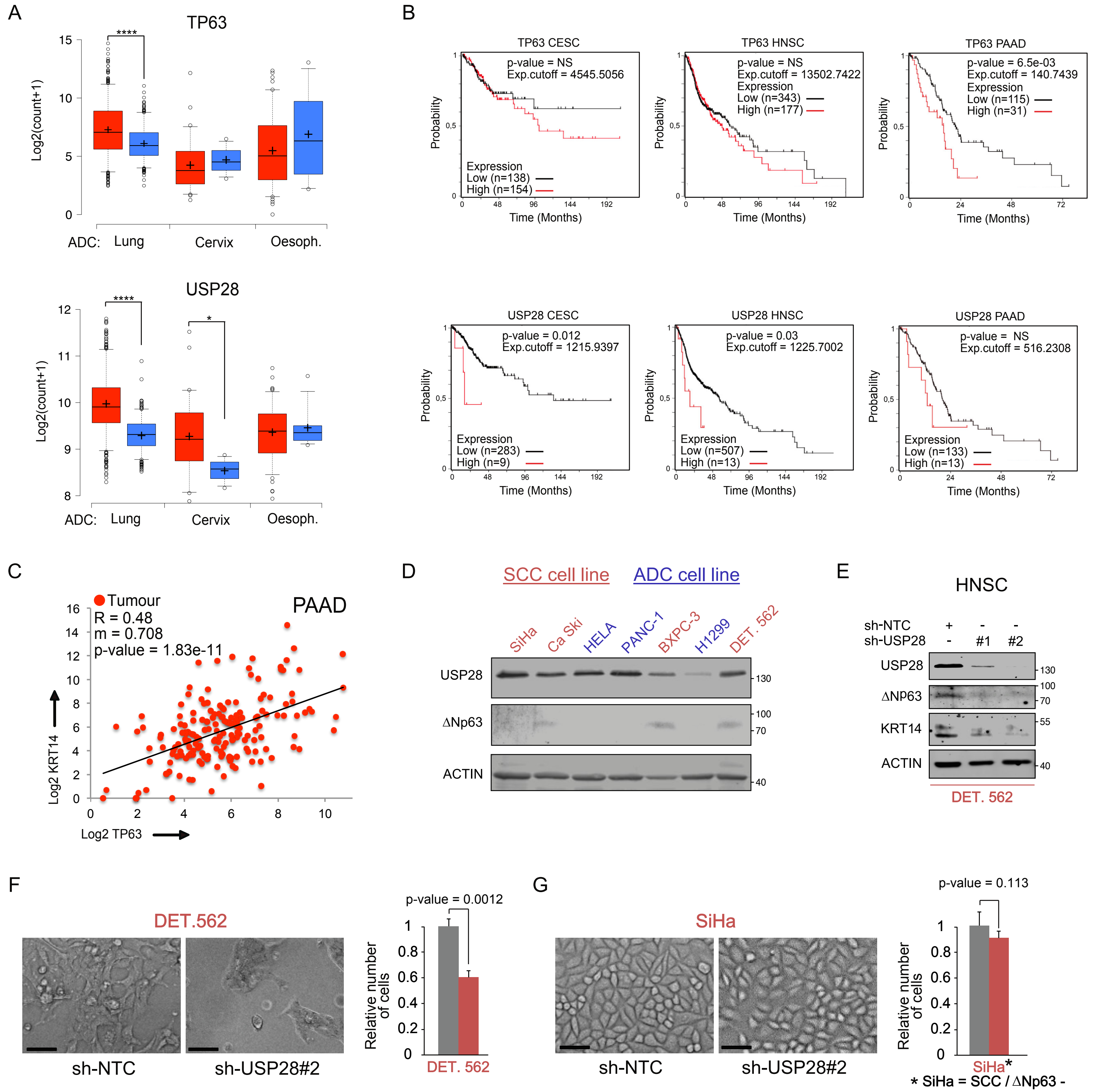

Appendix Figure S3: ΔNp63-driven SCC cells of various tissues are vulnerable to USP28 depletion.

A) Expression of TP63 and USP28 in human Lung (n=513), Cervix (n=47), and Oesophagus (n=89) ADC tumours and normal nontransformed tissue (nLung=348, nCervix=3, nOesophagus=11 and nHNSC=44). Xena UCSC. In the box plots, the centre line reflects the median, the cross represents the mean and the upper and lower box limits indicates the first and third quartile. Whiskers extend 1.5x the IQR. p-values were calculated using two-tailed t-test statistical analysis; *p<0.05 and ****p<0.001.

B) Kaplan-Meier estimator of patient survival in cervical SCC (CESC, n=292), head and neck SCC (HNSC, n=520) and pancreatic adenocarcinoma (PAAD, n=146) by USP28 and TP63 expression. Notably, only PAAD was significant for ΔNp63, indicating the SCC subtype. p-values were calculated using log-rank test. R2 software.

C) Correlation of mRNA expression of KRT14 and TP63 in PAAD tumours (n=146); R= Spearmans correlation coefficient; m= Slope.). Xena UCSC.

D) Immunoblot against endogenous USP28 and ΔNp63 in human CESC, PAAD, lung ADC and HNSC cancer cell lines. ACTIN served as loading control. Red= SCC and Blue= ADC; Notably, the human Cervix SCC cell line SiHa was negative for ΔNp63.

E) Immunoblot of USP28, ΔNp63 and KRT14 in HNSC cell line Detroit 562 transduced with non-targeting (sh-NTC) or two independent shRNA against USP28 (shUSP28#1 and #2). ACTIN served as loading control.

F) Brigthfield images of Detroit 562 were stably transduced with sh-NTC or shUSP28#2, seeded at equal cell density and counted after five days. Relative number of sh-USP28#2 cells compared with the sh-NTC control cells. p-values were calculated using two-tailed t-test statistical analysis. Scale bar = 30µm Quantitative graph is represented as mean ± SD of three experiments (n=3).

G) Brigthfield images of SiHa were stably transduced with sh-NTC or shUSP28#2, seeded at equal cell density and counted after five days. Relative number of sh-USP28#2 cells compared with the sh-NTC control cells. p-values were calculated using two-tailed t-test statistical analysis. Scale bar = 30µm. Quantitative graph is represented as mean ± SD of three experiments (n=3). SiHa* = Notably, the human Cervix SCC cell line SiHa was negative for ΔNp63.

Data information: All Immunoblots are representative of n=3. See also Appendix Table S3.

Appendix Table S1: Genes up and down regulated in shUSP28/shDNp63 vs shNTC.

shUSP28_Up-regulated	shUSP28_down-regulated
ADAM23	ACSS1
ADAMTS12	ADAD2
AEN	ADAMTS15
AGPAT4	ADAMTS16
ALDH1L2	ADD3
AMIGO2	ADGRF1
ARHGAP24	AGR2
ARHGEF26	AHNAK2
ARL14	AKR1B10
ARL4A	ALDH3A1
ATP2B1	ALDH3B2
BCHE	ALPG
BTBD19	ALPP
CEP85L	AMN
CNTD2	AMOT
COL3A1	AMY2B
COL5A2	ANK1
COL6A3	ANK3
CPED1	ANO1
CRELD2	ANPEP
ABCB5	ARHGAP33
CSF2	ARRB1
CSF3	ARRDC3
CXCL1	ASAP3
CXCL2	ATP2B4
CXCL3	BARX2
CXCL8	BCL11A
DBN1	BCL11B
DDIT3	BMF
DHRS2	BMP7
DMD	C4A
DNAJB11	C4B
DNAJB9	CA3-AS1
ETV5	CA9
FAT4	CALB2
FKBP4P6	CALR4P
FNIP2	CARD14
FSD1	CASP14
FST	CAVIN2
GADD45A	CCBE1
GDA	CCDC80
GDF15	CCL17
GEM	CCL22
GJA1	CCL28
GOLGA2P11	CD164L2
HBEGF	CD247
HDAC9	CD70

HEY1	CD74
HEY2	CD93
HMGA2	CDH23
HOXD1	CDKN1C
HOXD3	CEL
HSPA5	CELP
HTR7	CERS3
HYOU1	CERS4
IFI44L	CFI
IFI6	CIB4
IFNB1	CIITA
IFNL1	CKB
IGFL1	CLCA2
IL36G	CLEC7A
INHBE	CNFN
ISG15	COL12A1
KCNJ11	COLCA1
KIAA1549	CP
KLHL4	CPE
LIF	CRABP2
MANF	CRISPLD2
MIR22HG	AATK
MIR3142HG	CTSS
MT2A	CX3CL1
MT2P1	CYP2W1
MTRNR2L6	CYTH4
MX2	DAPK2
MYH16	DARS-AS1
NAV3	DCP1B
NEURL3	DEXI
NMRAL2P	DIP2C
NT5DC4	DNAJC27-AS1
NT5E	DNASE1L3
NUAK1	DOCK11
PANX2	DQX1
PAPPA2	EDN2
PDGFA	EFEMP2
PDIA4	EFNA3
PLK3	EGLN3
PORCN	EIF4E3
RADIL	ELF3
RBM24	EPB41L1
RIMS2	ERP27
RPS6KL1	ESPN
SDC3	EXOC3L4
SDF2L1	FA2H
SEC11C	FAM131C
SELENOKP3	FAM13A-AS1

SEMA3B	FAM178B
SEMA7A	FAM83E
SERPINB2	FAXDC2
SETBP1	FGFR3
SKIL	FLJ31356
SLC2A13	FOXA2
SLC7A11	FOXQ1
SMG7-AS1	FUT11
SPHK1	FUZ
SPP1	GASAL1
SPRY4	GBP2
TBC1D4	GGT6
TIGAR	GIPC2
TMEM171	GLIPR2
TMTC2	GLUL
TNFRSF12A	GLULP4
TNN	GNAO1
TRIM36	GOLGA8R
TRIML2	GPRC5C
TUSC3	GRID2IP
ZEB1	GRIP2
ZFP69B	GSDMC
ZNF438	GSTA4
ZNF704	H19
	HLA-DMB
	HLA-DPA1
	HLA-DPB1
	HLA-DRA
	HLA-DRB1
	HLA-DRB5
	HLA-DRB6
	HRK
	IGFBP3
	INPP5D
	IP6K3
	IRX3
	IZUMO4
	JPH2
	KCNC4
	KCNK3
	KCTD11
	KIAA1211L
	KLF8
	KLHL29
	KLK10
	KLK11
	KLK13
	KLK5

KLK6
KLRC4-KLRK1
KNDC1
KRT13
KRT14
KRT15
KRT16
KRT16P1
KRT16P2
KRT16P3
KRT16P4
KRT16P5
KRT16P6
KRT17P1
KRT17P2
KRT17P3
KRT17P6
KRT19
KRT3
KRT4
KRT5
KRT6A
KRT6B
KRT6C
KRT74
KSR1
KYNU
LDHD
LEMD1
LIMCH1
LRP1
LSP1
LYNX1
LYPD6
MAB21L3
MAGED1
MANCR
MAPK15
MB
MCAM
MEGF6
MESP1
MIR210HG
MITF
MMP7
MN1
MPP2
MPP7

MRC2
MST1
MUC1
MUC16
MUC20
MUC20P1
MXD4
MXI1
MYH14
MYLK4
MYO15B
MYO16
MYO1F
NDRG1
NEBL
NFATC4
NID1
NOD2
NOL4L
NOTCH3
NPAS3
NPNT
NRN1
NTN1
OBSCN
OLFM4
OLFML2A
OSBPL5
OSTN
PBX1
PCAT6
PCSK4
PEG10
PELI2
PGLYRP4
PIGR
PIK3IP1
PKIB
PLB1
PLIN4
PNPLA3
PNRC1
POF1B
POMT1
POU2AF1
PPFIA4
PPP1R3B
PREX1

PRICKLE1
PRKAR2A-AS1
PRODH
PTGES
PTPRH
RAB26
RAB3A
RAB40B
RALGDS
RBBP8NL
RBMXP4
RCSD1
REEP6
RGS16
RHOU
RHOV
RIPK4
RNF128
S100A4
S100A7
S100A8
S100A9
S100P
SCEL
SDCBP2
SECTM1
SEMA5B
SEMA6B
SERPINB4
SERPINF2
SGCB
SH2D3C
SH3BP2
SLC27A1
SLC27A2
SLC28A1
SLC29A3
SLC2A1-AS1
SLC47A2
SLIT2
SNHG28
SOWAHA
SPAG4
SPNS2
SPNS3
STAT4
STRC
SUGT1P1

SULF2
SUSD2
SYT15
SYT8
TBL1X
TCAF2
TGM1
THBS3
THEM5
THNSL2
TIAM1
TINCR
TJP3
TMC1
TMC4
TMEM156
TMEM45B
TMPRSS13
TMPRSS4
TNFRSF14-AS1
TNFSF10
TNNI2
TNNT3
TP53AIP1
TP73
TRIM17
TRIM29
TRPV4
TRPV6
TSPAN2
UNC5B
UNC5CL
UPK2
USH1G
USP28
VANGL2
VILL
VSTM5
VTCN1
WFDC10B
WFDC3
WFIKKN1
WISP1
WNT10A
WNT3A
WNT4
WNT9A
YPEL1

YPEL3
ZBTB7C
ZNF362
ZNF395
ZNF436-AS1
ZNF503
ZNF608
ZNF71

shDNP63_up-regulated	shDNP63_Down-regulated
ABI3BP	ACKR3
ACSM4	ADAD2
ACVRL1	ADAMTS15
ADAMTS12	ADGRF1
ADAMTS2	ADGRF4
ADAMTS7	ADGRG1
ADAMTSL1	AGR2
ADD3	ALDH1A1
ADORA1	ALDH3B2
AGPAT4	ALPK3
AGT	AMOT
AHRR	ANK1
AKR1B1	ANK3
AKR1B1P2	ANKRD22
AKR1C3	ANKRD65
AKT3	ANO1
ALPK2	ANO9
ANKRD1	ANPEP
ANKRD44	AQP3
APBA2	ARHGDIB
ARF4-AS1	ARHGEF16
ARHGAP22	ARL4C
ARHGEF26	ARRB1
ARL4A	ASB2
ARMCX1	ATF7IP2
ARX	BARX2
ASB9	BCL11A
ATOH8	BCL11B
ATP6V0A4	BHLHE41
ATRNL1	BICDL2
AXIN2	BSPRY
BLK	BX276092.7
BLZF2P	C16orf54
C12orf75	C1orf116
C1orf21	C1orf147
C1QTNF1	C1orf210
C3orf80	C6orf141
C4A	C9
C4A-AS1	CA12
C4B	CA2
C4B-AS1	CA3
CADPS2	CA3-AS1
CALHM3	CA5A
CAMK1D	CA9
CAPSL	CALB1
CARD11	CALB2
CAVIN3	CALML3-AS1

CBARP	CAMK2N1
CCDC190	CASP1
CD14	CASP14
CD22	CCDC197
CD33	CCL17
CD34	CCL22
CD8A	CCR7
CD99L2	CD164L2
CDH12P1	CD74
CDK15	CD93
CDON	CDC42EP5
CEMIP	CDH1
CEP85L	CDH23
CGA	CDH3
CHI3L1	CDIPTOSP
CLDN11	CEL
CMTM7	CELP
COL13A1	CERS3
COL18A1	CGB7
COL3A1	CIB4
COL5A3	CIITA
COL6A1	CISH
COL6A2	CKMT1B
COL6A3	CLCA2
COL8A1	CLEC7A
COLEC10	CMTM2
CORO2B	CNTNAP3
CPA4	CNTNAP3B
CPB1	COBLL1
CPEB4	COL17A1
CPLX1	COLCA1
CREB3L1	CORO1A
CRH	CP
ABCA3	CRABP2
CSF2	CREG2
CSF2RA	ACE2
CSF3	CRYBG2
CST7	CSTA
CTSO	CTSS
CXCL1	CX3CL1
CXCL2	CXADRP2
CXCL8	CXorf49
CYP21A2	CXorf49B
CYR61	CYP1A1
CYTIP	DAPP1
DBH	DARS-AS1
DBN1	DCP1B
DCLK2	DENND1C

DIO2	DENND2C
DKK1	DEXI
DMBT1	DLL1
DNER	DNASE1L3
DPF3	DOCK11
DPYSL4	DOK7
DUSP23	DSC2
DUSP8	DSC3
EBF4	DSG3
EBI3	DTX4
EDN1	EFNB1
EDNRB	EGLN3
EFR3B	ELF3
EGF	EMP1
EMP3	EPHA1
ENHO	EPN3
EPHA4	EPS8L1
EVA1A	ERP27
EXTL1	ESPN
F2R	ESRP1
FAM102B	EXOC3L4
FAM167A	FAM178B
FAM20C	FAM83B
FAM78B	FAT2
FCHO1	FBXL22
FLJ22447	FERMT1
FN1	FGD3
FOXD3-AS1	FGFBP1
FOXL2NB	FGFR3
FRMD4A	FOXA2
FRY	FOXQ1
FSD1	FUT3
FSIP2	FUT6
FSTL1	FXYD3
FYN	GALNT12
FZD9	GALNT3
GAS6-AS1	GALNT5
GCNT2	GBP6
GDF3	GGT6
GEM	GJB2
GFRA1	GLDC
GPR1	GPR37L1
GPR137C	GRHL1
GPR173	GRHL2
GPR55	GSDMC
GPSM1	H19
GRAMD1B	HAS3
GRIK4	HBG1

GUCY1B2	HES1
HAPLN3	HES2
HCG17	HIST1H1C
HCG18	HLA-DMB
HCN4	HLA-DRA
HECW1	HLA-DRB1
HNMT	HLA-DRB5
HOXD1	HLA-DRB6
HOXD3	HLA-V
IGFN1	HR
IL2RG	HS3ST1
IL32	HSD11B2
IL4I1	IGSF9
IL6	IL1RN
IL7R	IL22RA1
ILDR2	IL36RN
INHBB	IL6R
IQCN	INAVA
JAKMIP3	INPP5D
JSRP1	IP6K3
KAZN-AS1	IRAIN
KCNJ12	IRF6
KCNJ4	ITGB6
KLF2	IVL
KLF9	JAG1
KLHL4	JUP
KLKB1	KCNH4
L1CAM	KCNJ15
LGALS12	KCNJ5
LHX4	KCNK3
LIMCH1	KCP
LRIG1	KCTD12
LTF	KDF1
LZTS1	KIAA0040
MAL	KIAA1211L
MAP1B	KITLG
MAPK11	KLF5
MAPT	KLK10
MAT1A	KLK11
MEIS3	KLK13
MEX3B	KLK5
MGAT5B	KLK6
MGLL	KRT13
MIR3142HG	KRT14
MMP19	KRT15
MMP9	KRT16
MRAS	KRT16P2
MRGPRX3	KRT16P3

MSLN	KRT16P4
MTND6P22	KRT16P5
MUC12	KRT16P6
MVB12B	KRT17P1
MYADM	KRT17P2
MYBL1	KRT17P3
MYLKP1	KRT17P6
MYO18B	KRT19
MYPN	KRT3
NFAM1	KRT31
NKX6-1	KRT32
NLRP10	KRT4
NLRP3	KRT5
NOTUM	KRT6A
NOV	KRT6B
NUAK1	KRT6C
NXF3	KRT74
OGDHL	KRT79
OLFML2B	KRT9
OPRL1	KRTCAP3
OVCH2	KYNU
P2RX6P	LAD1
PANX2	LAMA5
PAPSS2	LCN2
PCBP3	LEXM
PDE4B	LSP1
PDE7B	LY6D
PHACTR3	LYPD6
PIEZO2	MAB21L3
PLA2G16	MAB21L4
PLA2G4C	MAGED1
PLAC8	MALL
PLEKHG4	MAP7
PLEKHG4B	MARVELD3
PLEKHS1	MB
PNUTL1	MCTP2
PODXL	MDFI
PPM1H	MEP1A
PREX1	MFNG
PRKAR2B	MIR205HG
PRLR	MIR9-3HG
PRR15	MMP7
PRSS12	MOB3B
PSG1	MPP7
PSG11	MPZL2
PSG2	MRPL23-AS1
PSG3	MUC16
PSG4	MUC20

PSG5	MUC20P1
PSG6	MYB
PSG7	MYH14
PSG8	NACAD
PSG9	NDRG1
PTPRR	NDUFA4L2
PVALEF	NECTIN1
RAB3IL1	NECTIN4
RAB9B	NEURL1
RANBP3L	NFASC
RAPSN	NFATC4
RARRES2	NGFR
RASA3	NOD2
RASD1	NOTCH3
RCN3	NPNT
REEP2	NTF4
RETREG1	NYNRIN
RFLNA	OLFM4
RGS4	OLFML2A
RGS9	OR2B6
RNF173	OSTF1
RNF174	OSTN
ROBO3	OTUB2
ROPN1B	OVOL1
ROR2	PADI3
RPH3AL	PAK6
RTN4RL2	PALM3
RUNX3	PALMD
SAMD11	PAX9
SARDH	PBX1
SAXO1	PCDH1
SCG2	PGLYRP4
SCN1B	PIGR
SDC3	PLA2G4F
SELENOM	PLB1
SEMA6B	PLEKHA7
SERTAD4-AS1	POF1B
SH3PXD2B	PPP4R4
SH3RF3	PRODH
SIPA1L2	PROM2
SIX2	PRSS8
SLC39A8	PRXL2A
SLC43A2	PTAFR
SLC4A4	PTK7
SLC5A4-AS1	PTPRU
SLFN12	RAB11FIP4
SLFN12L	RAB19
SMARCA1	RAB25

SMPD1	RAB38
SNPH	RASAL1
SOX6	RBBP8NL
SPARC	RGS16
SPATC1L	RHBG
SPEF2	RHOV
SPON2	RNF128
SPTLC3	RNF223
SRGN	RNVU1-18
STC1	RP1L1
SYDE1	RSAD2
SYNGR3	S100A14
SYT11	S100A7
SYTL4	S100A7A
TBC1D4	S100A8
TBC1D9	S100A9
TBXA2R	S100P
TDO2	SCEL
TENT5C	SDR42E1
TEX41	SEMA4A
TGFB2	SERPINB13
TGFB2-AS1	SERPINB4
TIMP2	SERPINB5
TMEM158	SH2D3C
TMEM37	SH3BP1
TMEM71	SH3PXD2A
TMPRSS3	SHROOM2
TNXA	SLC27A2
TPSAB1	SLC28A1
TPSB2	SLC7A8
TPTE2	SMARCA2
TRAF1	SNAI2
TWIST2	SOCS2
TXK	SPNS2
UAP1L1	ST14
UCP2	STAT4
VASH1	STOM
VCAM1	STON2
VIM	SULF2
VIM-AS1	SYT12
VPS37D	SYT8
VXN	TBL1X
WBP1LP2	TCAF2
WT1	TFCP2L1
ZBBX	TGM1
ZC3H12D	THBD
ZDHHC2	THEGL
ZEB1	TLL2

ZEB2	TMC4
	TMEM51-AS1
	TMEM79
	TMPRSS13
	TMPRSS4
	TNFSF15
	TNNI1
	TNNI2
	TNNT2
	TNNT3
	TNS4
	TP53AIP1
	TP63
	TPSP2
	TRPV6
	TSPAN1
	TSPAN13
	TTLL10
	UCA1
	UNC5B
	UPK2
	USH1G
	VANGL2
	VAV3
	VDR
	VGLL1
	VILL
	VIPR1
	VSNL1
	VSTM5
	VTCN1
	WFDC10B
	WNT10A
	WNT4
	WNT7A
	ZMYND15
	ZNF214
	ZNF503
	ZNF608
	ZNF71

Appendix Table S2 (page 21): Squamous markers.

Squamous Markers
ALOX15
ALOXE3
CALML3
CALML5
CLCA2
DSC3
DSG3
EPHA1
EPHA5
FAT2
FGFR2
FGFR3
FREM2
GLI1
IGFBP3
IL1A
IRF5
JUP
Klk10
KRT13
KRT13
KRT14
KRT15
KRT16
KRT19
KRT3
KRT34
KRT4
KRT5
KRT6A
KRT6B
KRT6C
KRT75
KRT84
MIR205HG
MMP10
MMP28
MMP7
NOTCH3
OVOL1
PERP
PGF
PKP1
PVRL1
PVRL4
RAB25
SERPINB13
SERPINB5
SOCS2
SOX2
SPRR1A
SPRR1B
TMPRSS13
TMPRSS3
TMPRSS4
TMPRSS5
TP63
TP63
TRIM29
TRIM7

Appendix Table S3 (page 22- 31): Exact P-values and statistical tests.

Fig1A		p-values	test
USP28			
SCC	Tumour vs Normal	8,12175E-53	Two tailed student t-test
ADC	Tumour vs Normal	1,08133E-21	Two tailed student t-test
SCC vs ADC		6,94807E-26	Two tailed student t-test
TP63			
SCC	Tumour vs Normal	4,65196E-91	Two tailed student t-test
ADC	Tumour vs Normal	6,67244E-06	Two tailed student t-test
SCC vs ADC		5,7528E-227	Two tailed student t-test

Fig1B		p-values	test
Spearman R2			
SCC	USP28 TP63	1,26E-112	Two tailed student t-test
ADC	USP28 TP63	8,21E-21	Two tailed student t-test

Fig1C		p-values	test
USP28			
	SCC vs ADC	0,005371154	Two tailed student t-test
	SCC vs SCLC	0,027031003	Two tailed student t-test
	SCC vs Normal	0,015366321	Two tailed T-test
			Two tailed student t-test
TP63			
	SCC vs ADC	0,005685114	Two tailed student t-test
	SCC vs SCLC	0,000951902	Two tailed student t-test
	SCC vs Normal	0,010045468	Two tailed student t-test

Fig1D		p-values	test
NSCLC survival			
	USP28	3,50E-07	Log-rank test
	TP63	1,20E-05	Log-rank test

Fig1E		p-values	test
SCC survival			
	USP28	0,0084	Log-rank test

Fig2F		p-values	test
TP63			
TP63 vs TP63+USP28	CHX hrs		
	4	0,010274653	Two tailed student t-test
	8	0,001093807	Two tailed student t-test
	12	4,86642E-05	Two tailed student t-test
TP63 + USP28 vs TP63 +USP28CA	CHX hrs		
	4	0,02725222	Two tailed student t-test
	8	0,002897423	Two tailed student t-test
	12	0,001967265	Two tailed student t-test
TP63 vs TP63+USP28CA	CHX hrs		
	4	0,214994093	Two tailed student t-test
	8	0,18510408	Two tailed student t-test
	12	0,873576497	Two tailed student t-test

Fig2F		p-values	test
TP63			
	PR619		
	1	0,844062024	Two tailed student t-test
	5	0,006409003	Two tailed student t-test
	10	0,001698956	Two tailed student t-test
	20	0,002461136	Two tailed student t-test
USP28			
	PR619		
	1	0,12307973	Two tailed student t-test
	5	0,006765345	Two tailed student t-test
	10	0,0016346	Two tailed student t-test
	20	0,004981223	Two tailed student t-test

Fig3D		p-values	test
TP63			
control vs sh-usp28	CHX		
	4	0,018420697	Two tailed student T-test
	8	0,047167383	Two tailed student T-test
	12	0,032685755	Two tailed student T-test
	24	0,298803036	Two tailed student T-test

Fig3G		p-values	test
TP63			
control vs mUSP28	CHX		
	4	0,075702468	Two tailed student T-test
	8	0,131690152	Two tailed student T-test
	12	0,018476643	Two tailed student T-test
	24	0,010728636	Two tailed student T-test

Fig4A		p-values	test
Pearson correlation			
	NTC/sh-Dnp63 vs NTCl/sh-USP28	<2,229E-308	Two tailed student t-test

Smallest number excel can calculate

Fig4B		p-values	test
Venn diagrams			
	NTC/sh-Dnp63 vs NTCl/sh-USP28		
	Up-regulated	3,99E-50	Hypergeometric test
	Down-regulated	2,19E-218	Hypergeometric test

GSEA=signal2Noise metric and 1000 permutations

Fig4D		p-values	test
GSEA			
	NTC vs sh-Dnp63	<1e-04	Nominal p value by GSEA
	NTC vs sh-USP28	<1e-04	Nominal p value by GSEA

Fig4E		p-values	test
GSEA			
	NTC vs sh-USP28	<1e-04	Nominal p value by GSEA
	NTC vs sh-Dnp63	<1e-04	Nominal p value by GSEA

Fig4F	p- value -log10	p-value	test
GO biological process Down	sh-Dnp63		
epidermis development	25,28903688	5,14E-26	Statistical overrepresentation test (Panther)
tissue development	24,42829117	3,73E-25	Statistical overrepresentation test (Panther)
epithelium development	22,23358715	5,84E-23	Statistical overrepresentation test (Panther)
skin development	20,28819277	5,15E-21	Statistical overrepresentation test (Panther)
cornification	19,41566878	3,84E-20	Statistical overrepresentation test (Panther)
epithelial cell differentiation	19,12378216	7,52E-20	Statistical overrepresentation test (Panther)
epidermal cell differentiation	17,56383735	2,73E-18	Statistical overrepresentation test (Panther)
keratinocyte differentiation	15,60730305	2,47E-16	Statistical overrepresentation test (Panther)
keratinization	12,36855623	4,28E-13	Statistical overrepresentation test (Panther)

Fig4G	p- value -log10	p-value	test
GO biological process Down	sh-Usp28	sh-usp28	
epidermis development	11,75696195	1,75E-12	Statistical overrepresentation test (Panther)
tissue development	10,90308999	1,25E-11	Statistical overrepresentation test (Panther)
skin development	10,47366072	3,36E-11	Statistical overrepresentation test (Panther)
keratinocyte differentiation	8,866461092	1,36E-09	Statistical overrepresentation test (Panther)
keratinization	7,07007044	8,51E-08	Statistical overrepresentation test (Panther)
epithelium development	10,35753548	4,39E-11	Statistical overrepresentation test (Panther)
epithelial cell differentiation	7,542118103	2,87E-08	Statistical overrepresentation test (Panther)
epidermal cell differentiation	9,931814138	1,17E-10	Statistical overrepresentation test (Panther)
cornification	10,32513886	4,73E-11	Statistical overrepresentation test (Panther)

Fig4I		p-values	test
Growth curve			
	day 7		
	ntc vs sh-USP28	0,014013748	Two tailed student t-test
	sh-USP28 vs sh-USP28 + Dnp63	0,005305166	Two tailed student t-test

Fig4M		p-values	test
GSEA Squamous Markers			
	NTC vs sh-Dnp63	<1e-04	Nominal p value by GSEA

Fig4N		p-values	test
GSEA Squamous Markers			
	NTC vs sh-USP28	<1e-04	Nominal p value by GSEA

Fig4O		p-values	test
Spearman Correlation			
	Squamous markers vs sh-Dnp63	2,20E-275	Two tailed student t-test

Fig4P		p-values	test
Spearman Correlation			
	Squamous markers vs sh-USP28	1,40E-44	Two tailed student t-test

Fig5D		p-values	test
%Tumor Area			
	kpl vs kplu tumours	0,009688967	Two tailed student t-test

Fig5E		p-values	test
Tumour number			
	kpl vs kplu	0,006751938	Two tailed student t-test

Fig5H		p-values	test
Survival Curve number			
	kpl vs kplu	0,0023	Log-rank test

Fig5N		p-values	test
%Tumor Area			
	kpl vs kplu tumours	8,55112E-05	Two tailed student t-test
Tumour number			
	kpl vs kplu	3,87305E-05	Two tailed student t-test

FIG6B		p-values	test
Tp63	SCC		
SCC	Lung	2,07757E-89	Two tailed student t-test
SCC	Cervix	3,56413E-07	Two tailed student t-test
SCC	Oesophagus	6,71839E-22	Two tailed student t-test
SCC	HNSC	2,76408E-08	Two tailed student t-test
USP28			
SCC	Lung	3,01673E-48	Two tailed student t-test
SCC	cervix	0,021	Two tailed student t-test

FIG6C		p-values	test
Krt14 tp63			
	Lung	1,86E-189	Two tailed student t-test
	cervix	1,08E-37	Two tailed student t-test
	Oesophagus	2,10E-39	Two tailed student t-test
Krt7 tp63			
	Lung	2,87E-115	Two tailed student t-test
	cervix	8,12E-09	Two tailed student t-test
	Oesophagus	4,74E-15	Two tailed student t-test

FIG6G		p-values	test
Rel Number of cells			
Lung	H1299 vs Ludlu adh	2,00E-04	Two tailed student t-test
cervix	Hela vs ca ski	4,70E-03	Two tailed student t-test
	si ha vs ca ski	2,30E-03	Two tailed student t-test
Pancreas	PANC1 vs BXPC3	1,90E-03	Two tailed student t-test

FIG 8D		p-value	test
control vs 125mg/kg		9,10872E-05	Two tailed student t-test
control vs 375 mg/kg		3,45892E-05	Two tailed student t-test
125mg/kg vs 375mg/kg		0,031	Two tailed student t-test

FIG 8F		p-value	test
control vs 125mg/kg		4,0683E-161	Two tailed student t-test
control vs 375 mg/kg		<2,229E-308	Two tailed student t-test
125mg/kg vs 375mg/kg		<2,229E-308	Two tailed student t-test

Smallest number excel can calculate

Appendix Figure 1F		p-values	test
TP63 S383A			
TP63 S383A vs TP63S383A+USP28	CHX hrs		
	4	0,067986874	Two tailed student t-test
	8	0,021643692	Two tailed student t-test
	12	0,001135674	Two tailed student t-test

Appendix Figure 1G		p-values	test
TP63 S383A			
	PR619		
	1	0,869609235	Two tailed student t-test
	5	0,011695422	Two tailed student t-test
	10	0,023529598	Two tailed student t-test
	20	0,003639534	Two tailed student t-test
USP28			
	PR619		
	1	0,069186507	Two tailed student t-test
	5	0,015959624	Two tailed student t-test
	10	0,018953154	Two tailed student t-test
	20	0,008878951	Two tailed student t-test

Appendix Figure2B		p-values	test
FBXW7			
	Lung	<0,005	Only range; Calculated by Gepia software (information in website and matheria/ methods)

Gepia p-value calculation for box plots = The differential analysis was based on: “TCGA tumours vs (TCGA normal + GTEx normal)”.. p-values were calculated with a one-way ANOVA comparing tumour with normal tissue.

Appendix Figure2H		p-values	test
TP63			
control vs sh-usp28	CHX		
	4	0,886705302	Two tailed student T-test
	8	0,000386309	Two tailed student T-test
	12	0,001229466	Two tailed student T-test
	24	0,104312972	Two tailed student T-test

Figure EV2B		p-values	test
Growth curve			
	day 7		
	ntc vs sh-DNP63 1	0,001786558	Two tailed student t-test
	ntc vs sh-DNP63 2	0,001101883	Two tailed student t-test

Figure EV2C		p-values	test
Cell cycle			
	s-phase		
	ntc vs sh-DNP63 1	0,030370454	Two tailed student t-test
	ntc vs sh-DNP63 2	0,04663474	Two tailed student t-test

Figure EV2D		p-values	test
Growth curve			
	day 7		
	ntc vs sh-USP28 1	0,001973272	Two tailed student t-test
	ntc vs sh-USP28 2	0,001199074	Two tailed student t-test

Figure EV2E		p-values	test
Cell cycle			
	s-phase		
	ntc vs sh-USP28 1	0,048779928	Two tailed student t-test
	ntc vs sh-USP28 2	0,041143341	Two tailed student t-test

Figure EV2G		p-values	test
GSEA			
	NTC vs sh-Dnp63	<1e-04	Nominal p value by GSEA

Figure EV2H		p-values	test
GSEA			
	NTC vs sh-USP28	<1e-04	Nominal p value by GSEA

Figure EV2J		p-values	test
GSEA			
	NTC vs sh-USP28		
	NOTCH1	0,4	Nominal p value by GSEA
	MYC	<1e-04	Nominal p value by GSEA
	AP1	0,035	Nominal p value by GSEA

Figure EV2K		p-values	test
GSEA			
	NTC vs sh-DNp63		
	NOTCH1	0,08	Nominal p value by GSEA
	MYC	1	Nominal p value by GSEA
	AP1	0,078	Nominal p value by GSEA

Figure EV3D		p-values	test
%Tumor Area			
	kp vs kpl tumours	0,037469688	Two tailed student t-test

Figure EV3E		p-values	test
survival			
	kp vs kpl	1,00E-03	Log-rank test

Figure EV3F		p-values	test
%Tumor Area			
	kpl ADC vs kplu tumours	0,012569321	Two tailed student t-test
	kpl SCC vs kplu tumours	0,003568175	Two tailed student t-test
	kpl ADC vs kpl SCC tumours	0,116237773	Two tailed student t-test

Appendix Figure3A		p-values	test
Tp63			
ADC	Lung	0,008685851	Two tailed student t-test
USP28			
ADC	Lung	1,32989E-15	Two tailed student t-test
ADC	Cervix	0,021	Two tailed student t-test

Appendix Figure3B		p-values	test
TP63 survival			
	CESC	5,88E-01	Log-rank test
	HNSC	6,06E-01	Log-rank test
	PAAD	6,50E-03	Log-rank test
USP28 survival			
	CESC	1,20E-02	Log-rank test
	HNSC	3,00E-02	Log-rank test
	PAAD	0,14	Log-rank test

Appendix Figure3C		p-values	test
Krt14 tp63			
	PAAD	1,83E-11	Two tailed student t-test

Appendix Figure3F		p-values	test
Rel Number of cells			
	DET.562 control vs AZ1	1,20E-03	Two tailed student t-test

Appendix Figure3G		p-values	test
Rel Number of cells			
	Si ha control vs sh-usp28	1,13E-01	Two tailed student t-test

<0,05 *

<0,01 **

<0,001 ***

<0,0001 ***

Appendix Table S4 (page 32): Antibody dilution list.

**Antibody**	**Company**	**Catalog Number**	**Species**	**Procedure**	**Dilution**
USP28	Sigma-Aldrich	HPA006778	Rabbit	WB	1/1000
	Sigma-Aldrich	HPA006779	Rabbit	IF/ IHC	1/100
USP25	Sigma-Aldrich	HPA018297	Rabbit	WB	1/1000
ACTIN/ B-ACTIN	Santa Cruz	sc-1616	Goat	WB	1/1000
	Santa Cruz	sc-47778	Mouse	WB	1/1000
VINCULIN	Sigma-Aldrich	V9131	Mouse	WB	1/1000
TUBULIN	Proteintech Europe	66031-1-lg	Mouse	WB	1/1000
HA	Abcam	16B12	Rabbit	WB	1/1000
FLAG	Sigma-Aldrich	F3165	Mouse	WB	1/1000
KRT5	Bimake	A5439	Rabbit	WB/IHC	1/1000 ; 1/100
	Santa Cruz	sc-66856	Rabbit	IHC	1/100
KRT14	Bimake	A5434	Rabbit	WB	1/1000
TTF1	Santa Cruz	sc-13040	Rabbit	IHC	1/100
P63/ ΔNP63	Biolegend	619002	Rabbit	WB	1/1000
	Bimake	A5182	Rabbit	IHC/IF	1/100
	Abcam	ab3239	Mouse	IF	1/100
	ThermoFisher	PA5-36069	Rabbit	WB	1/1000
GFP	Santa Cruz	sc-9996	Mouse	IHC	1/200
c-MYC	Santa Cruz	sc-764	Rabbit	WB	1/1000
Dylight anti rabbit 680	ThermoFisher	SA5-10042	Donkey	WB	1/10000
Dylight anti mouse 680	ThermoFisher	SA5-10170	Donkey	WB	1/10000
Dylight anti goat 680	ThermoFisher	SA5-10090	Donkey	WB	1/10000
Dylight anti rabbit 800	ThermoFisher	SA5-10044	Donkey	WB	1/10000
Dylight anti mouse 800	ThermoFisher	SA5-10172	Donkey	WB	1/10000
Dylight anti goat 800	ThermoFisher	SA5-10092	Donkey	WB	1/10000
Alexa 488 donkey anti rabbit	ThermoFisher	A21206	Donkey	IF	1/300
Alexa 488 donkey anti mouse	ThermoFisher	A21202	Donkey	IF	1/300
Alexa 555 donkey anti mouse	ThermoFisher	A10036	Donkey	IF	1/300

Appendix Table S5 (page 33-45): Consumables.

1st Antibodies	Company	Identifier	RRID
Polyclonal rabbit anti-USP28	Sigma-Aldrich	HPA006778	AB_1080520
Polyclonal rabbit anti-USP28	Sigma-Aldrich	HPA006779	AB_1080517
Polyclonal rabbit anti-USP25	Sigma-Aldrich	HPA018297	AB_1858672
Polyclonal rabbit anti-ACTIN/ B-ACTIN	Santa Cruz	sc-1616	AB_630836
Monoclonal mouse anti-ACTIN/ B-ACTIN (C4)	Santa Cruz	sc-47778	AB_626632
Monoclonal mouse anti-VINCULIN (hVIN-1)	Sigma-Aldrich	V9131	AB_477629
Monoclonal mouse anti-TUBULIN (1E4C11)	Proteintech Europe	66031-1-lg	AB_1104276 6
Monoclonal mouse anti-HA (16B12)	Abcam	ab130275	AB_1115688 4
Monoclonal mouse anti-FLAG (M2)	Sigma-Aldrich	F3165	AB_259529
Monoclonal rabbit anti-KRT5 recombinant mAb	Bimake	A5439	
Polyclonal rabbit anti-KRT5	Santa Cruz	sc-66856	AB_2249757
Monoclonal rabbit anti-KRT14 recombinant mAb	Bimake	A5434	
Polyclonal rabbit anti-TTF1 (H-190)	Santa Cruz	sc-13040	AB_793532
Polyclonal rabbit anti-TP63	Biolegend	619002	AB_2207170
Monoclonal rabbit anti-p63 recombinant	Bimake	A5182	
Polyclonal rabbit anti-p63	ThermoFisher	PA5-36069	AB_2553354
Monoclonal mouse anti-GFP (B-2)	Santa Cruz	sc-9996	AB_627695

Polyclonal rabbit anti-c-MYC (N-262)	Santa Cruz	sc-764	AB_631276

**2nd Antibodies**	**Company**	**Identifier**	**RRID**
Donkey anti-Mouse IgG (H+L) Cross-Adsorbed Secondary Antibody, DyLight 680	ThermoFisher	SA5-10170	AB_2556750
Donkey anti-Goat IgG (H+L) Cross-Adsorbed Secondary Antibody, DyLight 680	ThermoFisher	SA5-10090	AB_2556670
Donkey anti-Rabbit IgG (H+L) Cross-Adsorbed Secondary Antibody, DyLight 800	ThermoFisher	SA5-10044	AB_2556624
Donkey anti-Mouse IgG (H+L) Cross-Adsorbed Secondary Antibody, DyLight 800	ThermoFisher	SA5-10172	AB_2556752
Donkey anti-Goat IgG (H+L) Cross-Adsorbed Secondary Antibody, DyLight 800	ThermoFisher	SA5-10092	AB_2556672
Donkey anti-Rabbit IgG (H+L) Highly Cross-Adsorbed Secondary Antibody, Alexa Fluor 488	ThermoFisher	A21206	AB_2535792
Donkey anti-Mouse IgG (H+L) Highly Cross-Adsorbed Secondary Antibody, Alexa Fluor 488	ThermoFisher	A21202	AB_141607
Donkey anti-Mouse IgG (H+L) Highly Cross-Adsorbed Secondary Antibody, Alexa Fluor 555	ThermoFisher	A31570	AB_2536180

Bacterial Strains	Company	Identifier
**DH5α** F- endA1 glnV44 thi-1 recA1 relA1 gyrA96 deoR nupG Φ80dlacZΔM15 Δ(lacZYA-argF)U169, hsdR17(rK-mK+), λ–	ThermoFisher	18263012
**Chemicals and Commercial Assays**	**Company**	**Identifier**
Gibco™ Dulbecco's Modified Eagle Medium (DMEM), high glucose	ThermoFisher	11574486
Gibco™ RPMI 1640 Medium	ThermoFisher	21875158
Gibco™ Trypsin-EDTA (0.5%), No Phenol Red	ThermoFisher	15400054
Fetal Bovine Serum (FCS)	Sigma-Aldrich	12103C
Penicillin-Streptomycin	Sigma-Aldrich	P4333
Gibco™ GlutaMAX™ Supplement	ThermoFisher	35050061
Polybrene	Sigma-Aldrich	TR-1003
Polyethylenimine, Linear, MW 25000, Transfection Grade (PEI 25K)	Polysciences	23966-1
Dimethyl sulfoxide (DMSO)	Sigma-Aldrich	D8418
Ethanol (Etoh)	Carl Roth	5054.6
Gibco™ Phosphate-buffered saline (PBS)	ThermoFisher	10010031
Nuclease-free water	Merck	3098
Cycloheximide	Sigma-Aldrich	1810
Doxycycline hyclate	Sigma-Aldrich	D9891
Tandem ubiquitin binding entity (TUBE)	Homemade	
PR-619	Selleckchem	S7130
AZ1	Probechem	PC-60023
Propidium Iodide (PI)	Sigma-Aldrich	11348639001
Protease Inhibitor Cocktail	Roche	4693159001
Pierce™ Protein A/G Magnetic Beads	ThermoFisher	88802
RIPA Lysis Buffer	Homemade	
4′,6-diamidino-2-phenylindole (DAPI)	ThermoFisher	D1306
Hoechst	ThermoFisher	62249
Coomassie Brilliant Blue R-250 Dye	ThermoFisher	20278
Phosphate-buffered saline (PBS)	Homemade	
2-Propanol/ Isopropanol	ROTH	AE73.2
Adenosintriphosphat (ATP)	Jena Bioscience	NU-1010-10G
Agarose	ROTH	3810.4
Ampicillin (Amp)	ROTH	HP62.2
Bovine serum albumine (BSA)	Merck Millipore	810683
Mowiol® 40-88	Sigma-Aldrich	324590
Dithiothreitol (DTT)	Sigma-Aldrich	D9779
Eosin	Sigma	E4009
Hematoxylin	Sigma	H3136
Polyvinylidene difluoride membranes (PVDF) Immobilon Transfer Membrane	Merck	IPFL00010
N,N,N',N'-tetramethylenethylendiamine (TEMED)	ROTH	2367.3
Natrium chloride (NaCl)	AppliChem	A2942,1000

Neutrally buffered formalin (NBF)	Thermo Fisher	5700TS
Tris-HCl	ROTH	9090.5
TritonX100	ROTH	3051.3
Xylene	Sigma	534056
β-Mercaptoethanol	ROTH	4227.1
Methanol (MeOH)	ROTH	0082.3
Transfer Buffer (Westerm Blot)	Homemade	
Blocking Buffer (Western Blot)	Homemade	
Wash Buffer (western Blot(	Homemade	
SDS Loading Buffer/ lämmli buffer (5X)	Homemade	
SDS Running Buffer (Western Blot)	Homemade	
Primary antibody buffer (Western Blot)	Homemade	
Secondary antibody buffer (western Blot)	Homemade	
peq GOLD Trifast	VWR (Peqlab brand)	30-2010
M-MLV reverse transcriptase	Promega	M1701
M-MLV RT 5X Buffer	Promega	M531A
Deoxynucleotidetriphosphates (dNTPs) Mix	Promega	U151A
Random Hexamer Primer	ThermoFisher	SO142
RiboLock RNase Inhibitor	ThermoFisher	EO0381
SYBR™ Green PCR Master Mix	ThermoFisher	4309155
ReliaPrep™ RNA Cell Miniprep System Protocol	Promega	TM370
NEBNext® Ultra™RNA Library Prep Kit for Illumina	New England Biolabs (NEB)	NEB #E7530S
NEBNext® Multiplex Oligos for Illumina® (Dual Index Primers Set 1)	New England Biolabs (NEB)	NEB #E7600S
NEBNext® Poly(A) mRNA Magnetic Isolation Module	New England Biolabs (NEB)	NEB #E7490S
**Cell lines**	**Company**	**Identifier**
Human: HEK 293T	ATCC	ATCC® CRL-11268™
Human: A-431	ATCC	ATCC® CRL-1555
Human: LUDLU-1	ECACC	92012463
Human: H1299	ATCC	ATCC® CRL-5803
Human: HELA	ATCC	ATCC® CCL-2
Human: SiHa	ATCC	ATCC® HTB-35
Human: Ca Ski	ATCC	ATCC® CRL-1550
Human: PANC-1	ATCC	ATCC® CRL-1469

Human: BXPC-3	ATCC	ATCC® CRL-1687
Human: Detroit 562	ATCC	ATCC® CCL-138
Mouse: KP ADC	Primary tumours	
Mouse: KP SCC	Primary tumours	
**Experimental Models: Organisms/Strains**	**Company**	**Identifier**
B6(C)-Gt(ROSA)26Sor$^{em1.1(CAG-cas9*,-EGFP)Rsky}$/J	The Jackson laboratory	Stock No: 028555
B6.129-Kras^{tm4Tyj} Trp53^{tm1Brn}/J	The Jackson laboratory	Stock No: 032435
C57BL/6J	The Jackson Laboratory	Stock No: 000664
**Oligonucleotides**	**Sequence**	**Company**
hUsp28 FW	ACTCAGACTATTGAACAGATGTACTGC	Sigma
hUsp28 RV	CTGCATGCAAGCGATAAGG	Sigma
hKeratin10 FW	GCAAATTGAGAGCCTGACTG	Sigma
hKeratin10 RV	CAGTGGACACATTTCGAAGG	Sigma
hB-Actin FW	GCTACGAGCTGCCTGACG	Sigma
hB-Actin RV	GGCTGGAAGAGTGCCTCA	Sigma
hGpcr5A FW	AAGGTCTCCCCCAGCACT	Sigma
hGpcr5A RV	GGGACTGTTGTAGCCATTCTG	Sigma
hTp63 FW	GGAAAACAATGCCCAGACTC	Sigma
hTp63 RV	GTGGAATACGTCCAGGTGGC	Sigma
hTp63-2 FW	GAAAGCTGTTCCTTGGTCCTAGT	Sigma
hTP63-2 RV	GGTTTATTCAAACCCTCAGCA	Sigma
hKeratin5 FW	TCACCGTTCCTGGGTAACA	Sigma
hKeratin5 RV	GGAGGTGCTGGAGAGAACAG	Sigma
hKeratin14 FW	GGAAGTGAAGATCCGTGACTG	Sigma
hKeratin14 RV	GGACTGTAGTCTTTGATCTCAGCA	Sigma
hKeratin19 FW	TTGTCCTGCAGATCGACAAC	Sigma

hKeratin19 RV	GCCTGTTCCGTCTCAAACTT	Sigma
mSftpc5 FW	GGTCCTGATGGAGAGTCCAC	Sigma
mSftpc5 REV	GATGAGAAGGCGTTTGAGGT	Sigma
mkeratin5 FW	CAGAGCTGAGGAACATGCAG	Sigma
mKeratin5 RV	CATTCTCAGCCGTGGTACG	Sigma
mScgb1a1 FW	TTGTCACTGCCCTGTGTCTC	Sigma
mScgb1a1 REV	AAGAGGAAGGAGGGGTTGG	Sigma
msox2 FW	GGCAGAGAAGAGAGTGTTTGC	Sigma
mSox2 REV	TCTTCTTTCTCCCAGCCCTA	Sigma
mUSP28 FW	ATGACAACTTGCCCCACTTC	Sigma
mUSP28 RV	AGTTCCACAGACAGGGCTTC	Sigma
mB-Actin FW	AGTGTGACGTTGACATCCGT	Sigma
mB-Actin RV	TGCTAGGAGCCAGAGCAGTA	Sigma
human shRNA ΔNp63 #1 for	TGAATGAACAGACGTCCAATTTCTCGAGAAATTGGACGTCTGTTCATTCTTTTTC	Sigma
human shRNA ΔNp63 #1 rev	TCGAGAAAAAGAATGAACAGACGTCCAATTTCTCGAGAAATTGGACGTCTGTTCATTCA	Sigma
human shRNA ΔNp63 #2 for	TCGAGTGGAATGATTTCAACTTCTCGAGAAGTTGAAATCATTCCACTCGTTTTTC	Sigma
human shRNA ΔNp63 #2 rev	TCGAGAAAAACGAGTGGAATGATTTCAACTTCTCGAGAAGTTGAAATCATTCCACTCGA	Sigma

human shRNA USP28 #1 for	CCGGCAAGGAGCTTATTCGAAATCTCGAGATTTCGAATAAGCTCCTTGTTTTTG	Sigma
human shRNA USP28 #1 rev	AATTCAAAAACAAGGAGCTTATTCGAAATCTCGAGATTTCGAATAAGCTCCTTG	Sigma
human shRNA USP28 #2 for	CCGGGACTGAAGATCATCCATTACTCGAGTAATGGATGATCTTCAGTCTTTTTG	Sigma
human shRNA USP28 #2 rev	AATTCAAAAAGACTGAAGATCATCCATTACTCGAGTAATGGATGATCTTCAGTC	Sigma
murine shRNA USP28 #1 for	TGCTGTTGACAGTGAGCGAGGATGTGAATTTGTATAAAAATAGTGAAGCCACAGATGTATTTTTATACAAATTCACATCCCTGCCTACTGCCTCGGA	Sigma
murine shRNA USP28 #2 for	TGCTGTTGACAGTGAGCGATCTGTTTATACTTTAGATAAATAGTGAAGCCACAGATGTATTTATCTAAAGTATAAACAGACTGCCTACTGCCTCGGA	Sigma
sgRNA murine Stk11/Lkb1 for	CACCGCGAGACCTTATGCCGCAGGG	Sigma
sgRNA murine Stk11/Lkb1 rev	AAACCCCTGCGGCATAAGGTCTCGc	Sigma
sgRNA murine Usp28 #1 for	CACCGGGGAGCCTTCCGATCATCCG	Sigma
sgRNA murine Usp28 #1 rev	AAACCGGATGATCGGAAGGCTCCCc	Sigma
sgRNA murine Usp28 #2 for	CACCGCGGATCGTTCCGTGAAGTAT	Sigma
sgRNA murine Usp28 #2 rev	AAACATACTTCACGGAACGATCCGc	Sigma

sgRNA murine Kras #1 for	CACCGACTGAGTA TAAACTTGTGG	Sigma
sgRNA murine Kras #1 rev	AAACCCACAAGTT TATACTCAGTC	Sigma
sgRNA murine Trp53 #1 for	CACCGATGGTGG TATACTCAGAGC	Sigma
sgRNA murine Trp53 #1 rev	AAACGCTCTGAGT ATACCACCATC	Sigma
FLAG-deltaNp63alpha S383A Sited-Directed mutagenesis for	CAGCATGAACAAG CTGCCTGCCGTG AGCCAGCTTATCA ACCCAC	Sigma
FLAG-deltaNp63alpha S383A Sited-Directed mutagenesis rev	GTGGGTTGATAAG CTGGCTCACGGC AGGCAGCTTGTTC ATGCTG	Sigma
KrasG12D repair template for	TTTTGTGTAAGCT TTGGTAACTCCAT GTATTTTTATTAAG TGTT	Sigma
KrasG12D repair template rev	GAGCTTATCGATA CCGTCGACACAC CCAGTTTAAAGCC TTGGAA	Sigma
**Recombinant DNA**	**Company/Source**	**Identifier**
pLKO.DEST.EGFP	pLKO.DEST.EGFP was a gift from Ming-Sound Tsao (Addgene plasmid # 32684 ; http://n2t.net/addgene:32684 ; RRID:Addgene_32684)	Addgene plasmid # 32684
pLKO.1 puro	pLKO.1 puro was a gift from Bob Weinberg (Addgene plasmid # 8453 ; http://n2t.net/addgene:8453 ; RRID:Addgene_8453)	Addgene plasmid # 8453

pINDUCER20	pInducer20 was a gift from Stephen Elledge (Addgene plasmid # 44012 ; http://n2t.net/addgene:44012 ; RRID:Addgene_44012)	Addgene plasmid # 44012
deltaNp63alpha-FLAG	deltaNp63alpha-FLAG was a gift from David Sidransky (Addgene plasmid # 26979 ; http://n2t.net/addgene:26979 ; RRID:Addgene_26979)	Addgene plasmid # 26979
deltaNp63alpha S383A-FLAG	This publication	N/A
pLKO-eGFP-shdeltaNp63-1 (GFP)	This publication	N/A
pLKO-eGFP-shdeltaNp63-2 (GFP)	This publication	N/A
pDZ Flag USP28	pDZ Flag USP28 (Addgene plasmid # 15665 ; http://n2t.net/addgene:15665 ; RRID:Addgene_15665)	Addgene plasmid # 15665
pINDUCER20 -mouse Usp28 WT (Neomycin)	This publication	N/A
pLKO shUSP_28_1 (Puromycin)	This publication	N/A
pLKO shUSP_28_2 (Puromycin)	This publication	N/A
pcDNA3-HA-USP28	pcDNA3-HA-USP28 was a gift from Nikita Popov	N/A
pcDNA3-HA-USP28 C171A	pcDNA3-HA-USP28C171A was a gift from Nikita Popov	N/A
deltaNp63alphaS383A-FLAG	Site directed mutagenesis in deltaNp63alpha-FLAG	N/A
pGEPIZ 20-human-sh-Usp28 (Neomycin)	pINDUCER 20-human-sh- Usp28 was a gift from Carina Maier (Almut Schulze group)	N/A
pcDNA3 His6-Ubi K48	pcDNA3 His6-Ubi K48 was a gift from Amir Orian	N/A

pcDNA3 His6-Ubi K63	pcDNA3 His6-Ubi K63 was a gift from Amir Orian	N/A
pcDNA3 His6-Ubi	pcDNA3 His6-Ubi was a gift from Amir Orian	N/A
psPAX2	psPAX2 was a gift from Didier Trono (Addgene plasmid # 12260 ; http://n2t.net/addgene:12260 ; RRID:Addgene_12260)	Addgene plasmid # 12260
pMD2G	pMD2.G was a gift from Didier Trono (Addgene plasmid # 12259 ; http://n2t.net/addgene:12259 ; RRID:Addgene_12259)	Addgene plasmid # 12259
pHelper	Cell Biolabs, INC.	VPK-400-DJ
pAAV2/8	AAV2/8 was a gift from James M. Wilson (Addgene plasmid # 112864 ; http://n2t.net/addgene:112864 ; RRID:Addgene_112864)	Addgene plasmid # 112864
pAAV-DJ Vector	Cell Biolabs, INC.	VPK-420-DJ
pLKO-eGFP-mshUSP_28_1 (GFP)	This Paper	N/A
pLKO--eGFP- mshUSP_28_2 (GFP)	This Paper	N/A
AAV:ITR- U6-sgRNA(p53)- pEFS-2A-mCherry-shortPA- ITR	This publication	N/A
AAV:ITR-U6-sgRNA(Kras)-pEFS-2A-mCherry-shortPA-KrasG12D_HDRdonor-ITR	This publication	N/A
AAV:ITR-U6-sgRNA(Kras)-U6-sgRNA(p53)-pEFS-2A-mCherry-shortPA-KrasG12D_HDRdonor-ITR	This publication	N/A
AAV:ITR-U6-sgRNA(Kras)-U6-sgRNA(p53)-U6-sgRNA(Lkb1)- pEFS-2A-mCherry-shortPA-KrasG12D_HDRdonor-ITR	This publication	N/A

AAV:ITR-U6-sgRNA(Kras)-U6-sgRNA(p53)-U6-sgRNA(Lkb1)-U6-sgRNA(Usp28[1])-U6-sgRNA(Usp28[2])-pEFS-2A-mCherry-shortPA-KrasG12D_HDRdonor-ITR	This publication	N/A
AAV:ITR-U6-sgRNA(Kras)-U6-sgRNA(p53)-U6-sgRNA(Lkb1)-pEFS-Rluc-2A-Cre-shortPA-KrasG12D_HDRdonor-ITR (AAV-KPL)	AAV:ITR-U6-sgRNA(Kras)-U6-sgRNA(p53)-U6-sgRNA(Lkb1)-pEFS-Rluc-2A-Cre-shortPA-KrasG12D_HDRdonor-ITR (AAV-KPL) was a gift from Feng Zhang (Addgene plasmid # 60224 ; http://n2t.net/addgene:60224 ; RRID:Addgene_60224)	Addgene plasmid # 60224
**Software and Algorithm**	**Company/Source**	
cBioportal	https://www.cbioportal.org	
GEPIA	http://gepia.cancer-pku.cn	
KM-plotter	http://kmplot.com/analysis/	
BoxPlotR	http://shiny.chemgrid.org/boxplotr/	
Excel	Microsoft	
Venn diagrams	http://bioinformatics.psb.ugent.be/webtools/Venn/	
Image Studio	Licor	
Panther Classification system	http://pantherdb.org	
AATBIO IC50 calculator	https://www.aatbio.com/tools/ic50-calculator	
GraphPad Software	GraphPad Software, Inc.	
Affinity Designer	Serif Europe	
ImageJ	National Insistute of Health	
Primerx	http://www.bioinformatics.org/primerx/cgi-bin/DNA_1.cgi	
Pannoramic Case Viewer	3dHistech	
R2: Genomics Analysis and Visualization Platform	http://r2.amc.nl	

UCSC Xena	https://ucsc-xena.gitbook.io/project/	
GenerateFastq v1.1.0.64	http://emea.support.illumina.com/downloads/local-run-manager-generate-fastq-module.html	
FastQC	http://www.bioinformatics.babraham.ac.uk/projects/fastqc/	
Bowtie2 v2.3.4.1	http://bowtie-bio.sourceforge.net/index.shtml	
TopHat v.2.1.1	https://ccb.jhu.edu/software/tophat/index.shtml	
Samtools v1.3	http://samtools.sourceforge.net	
R	https://www.r-project.org	
EdgeR	https://bioconductor.org/packages/release/bioc/html/edgeR.html	
GenomicAlignments	https://bioconductor.org/packages/release/bioc/html/GenomicAlignments.html	
GSEA v2.2	http://software.broadinstitute.org/gsea/downloads.jsp	
**Instrument**	**Company/Source**	
Odyssey® CLx Imaging System	Licor	
iBright™ FL1000 Imaging System	Invitrogen	
BD FACSCanto II Cell Analyzer	BD Biosciences	
StepOnePlus Real-Time PCR System	ThermoFisher	
Invitrogen Countess II FL Automated Cell Counter	ThermoFisher	
Pannoramic DESK scanner	3DHISTECH	
FSX100 microscopy	Olympus Life Science	
Operetta screening and imaging system	Perkin Elmer	
Fragment Analyzer	Agilent formerly Advanced Analytical	
Axiocam 503 mono + Zeiss axio microscope	Zeiss	
Branson Sonifier 250	Branson	
Hyrax M55 Rotary Microtome	Leica	
PCR cycler: SimpliAmp thermo cycler	Life technologies	
