# Supplementary material for: Maintaining protein stability of ∆Np63 via USP28 is required by squamous cancer cells
Source: EMBO Mol Med. 2020 Mar 4;12(4):e11101. doi: 10.15252/emmm.201911101 (PMC7136964; doi:10.15252/emmm.201911101)
Supplement: Supplementary file 2 — Expanded View Figures PDF [file EMMM-12-e11101-s002.pdf]

Expanded View Figures

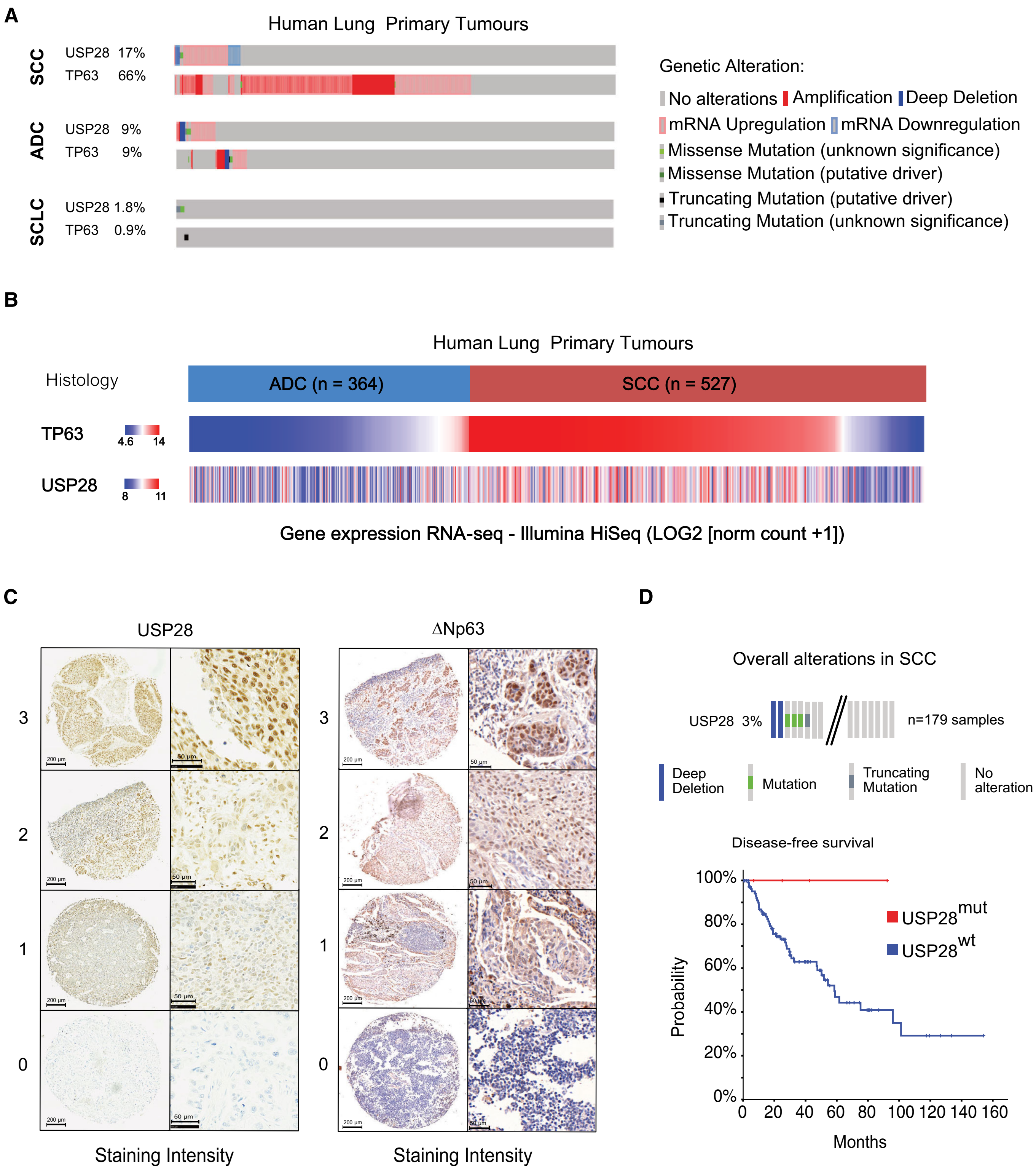

Figure EV1.

© 2020 The Authors

◀

Figure EV1. USP28 and ΔNp63 mRNA and protein expression in public datasets, TMA and patient material.

A Analysis of occurring genetic alterations in USP28 and TP63 in lung cancer (CBioPortal).

B USP28 and TP63 gene expression heatmap in ADC (n = 364) and SCC (n = 527) lung cancer samples (Xena UCSC software).

C Representative IHC grading scores of endogenous USP28 and ΔNp63 in lung tissue samples (left panel, low magnification, scale bar 200 μm; right panel high magnification, scale bar 50 μm).

D Genetic alterations of USP28 in human lung SCC. Each column represents a tumour sample (n = 179 LSCC). Disease-free survival of USP28 mutant lung SCC patients. Data from TCGA were analysed using cBioPortal software.

Figure EV2. SCC tumour cells are dependent on USP28 and/or ΔNp63 to maintain a SCC identity.

▶

A Immunoblot of endogenous ΔNp63 and USP28 in A-431 cells stably transduced with shRNA-non-targeting control (NTC) and two shRNA against ΔNp63. Actin served as loading control. n = 3.

B Cell growth of A-431 cells stably transduced with shRNA-non-targeting control (NTC) and two shRNA against ΔNp63. Total cell number was measured in triplicate and assessed at indicated time points.

C Cell cycle profile analysis by propidium iodide staining of stable ΔNp63 knock-down A-431 cells by two independent shRNA sequences. n = 3.

D Immunoblot of endogenous ΔNp63 and USP28 in A-431 cells stably transduced with shRNA-non-targeting control (NTC) and two shRNA against USP28. ACTIN served as loading control. n = 3.

E Cell growth of A-431 cells stably transduced with shRNA-non-targeting control (NTC) and two shRNA against USP28. Total cell number was measured in triplicate and assessed at indicated time points.

F Cell cycle profile analysis by propidium iodide staining of stable USP28 knock-down A-431 cells by two independent shRNA sequences. n = 3.

G Gene set enrichment analyses of USP28#1-silenced A-431 cells compared to shRNA-NTC using the gene list: "Genes Up-regulated sh-ΔNp63". NES, normalized enrichment score; $P < 0.0001$.

H Gene set enrichment analyses of ΔNp63-silenced A-431 cells compared to shRNA-NTC using the gene list: "Genes Up-regulated sh-USP28". NES, normalized enrichment score; $P < 0.0001$.

I Relative expression of consensus markers for squamous cancer, as used in Fig 4, in a pan-cancer panel (GEPIA software).

J Gene set enrichment analyses of USP28-silenced A-431 cells compared to shRNA-NTC using the gene list: "Hallmark NOTCH Signaling", "Hallmark MYC targets V1" and "PID AP1 Pathway". NES, normalized enrichment score.

K Gene set enrichment analyses of ΔNp63-silenced A-431 cells compared to shRNA-NTC using the gene list: "Hallmark NOTCH Signaling", "Hallmark MYC targets V1" and "PID AP1 Pathway". NES, normalized enrichment score.

Data information: All quantitative data are represented as mean ± SD; *$P < 0.05$, **$P < 0.01$. Two-tailed t-test. See also Appendix Table S3 (exact P-values and statistical test used).

© 2020 The Authors

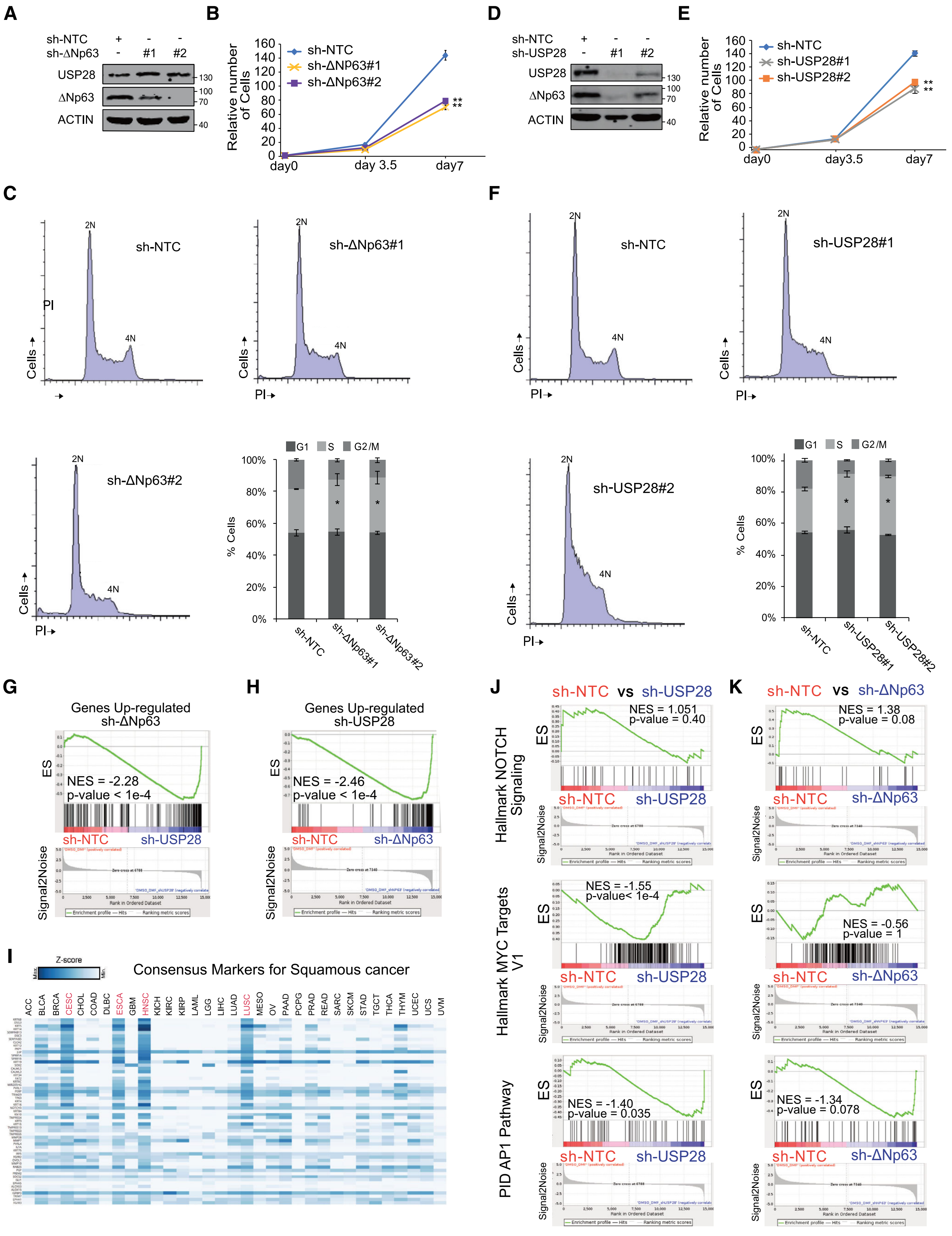

Figure EV2.

© 2020 The Authors

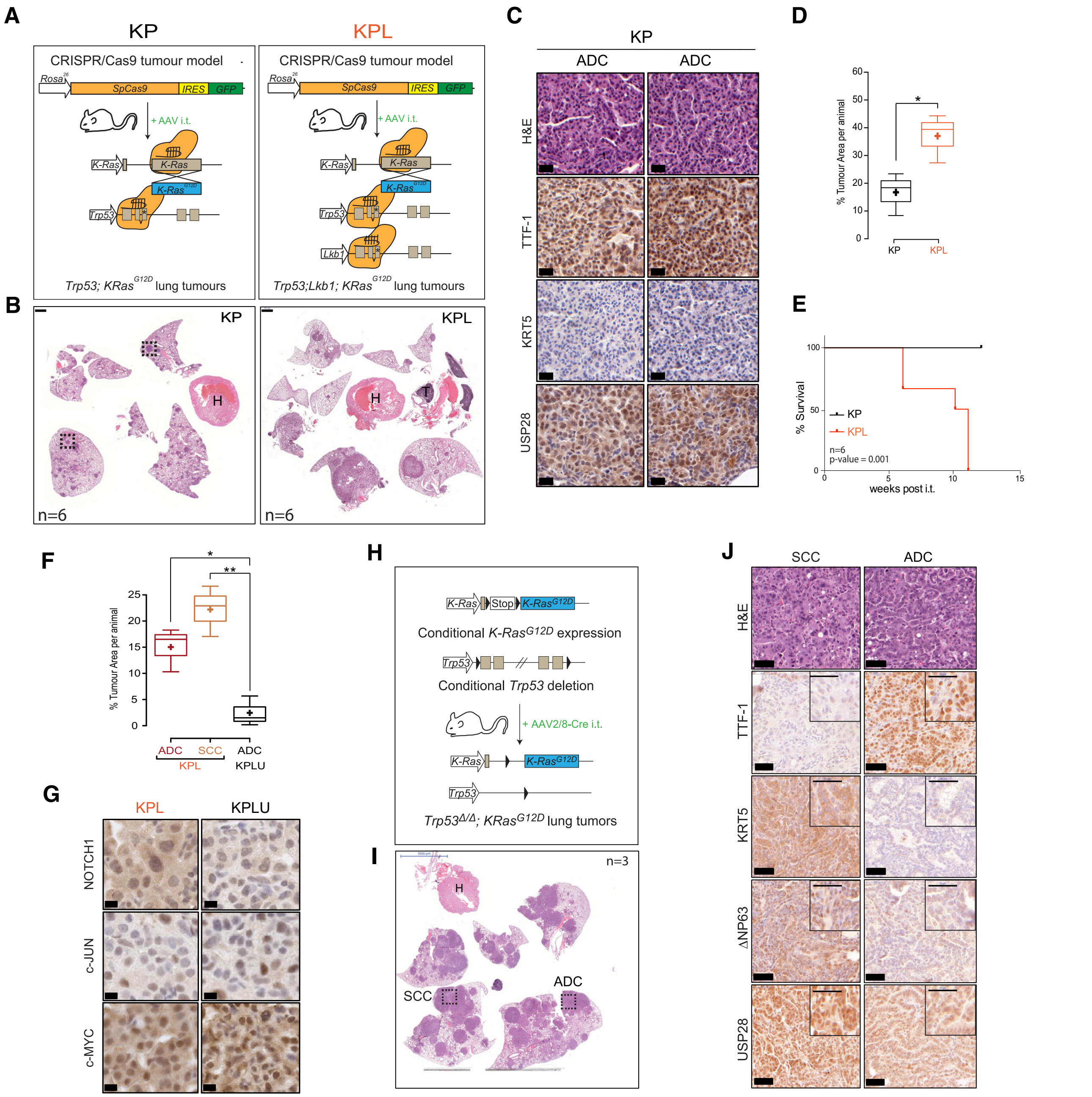

Figure EV3. Establishing and characterizing SCC mouse models.

A Schematic diagram of CRISPR/Cas9-mediated tumour modelling and targeting of p53 and KRasG12D(KP) or p53; LKB1 and KRasG12D(KPL) mouse lines.

B Representative H&E images of tumour-bearing animals 12 weeks post-intratracheal infection. Boxes indicate individual tumour areas assessed by IHC against marker proteins and USP28 (H = heart, T = thymus, scale bar: 1,000 μm); *n* = 6.

C IHC analysis of ADC and SCC marker expression, as well as USP28 abundance, in KP and KPL lung tumours (scale bar: 20 μm); *n* = 3.

D Box plot analysis of % tumour area and in KP and KPL animals; *n* = 6.

E Kaplan–Meier plot of comparing KP versus KPL animals (log-rank test, *P* = 0.001; *n* = 6).

F Quantification of SCC and ADC % tumour area (normalized to total lung area) in KPL (*n* = 6) and KPLU (*n* = 5) animals.

G IHC analysis of NOTCH1, c-MYC and c-JUN in KPL and KPLU lung tumours (scale bar: 20 μm).

H Schematic diagram of the classic KP mouse model (p53 fl/fl; lsl-KRasG12D).

I Representative H&E images of tumour-bearing animals 12 weeks post-intratracheal infection (H = heart; scale bar: 5000 μm); *n* = 3.

J IHC analysis of ADC and SCC marker expression, as well as USP28 abundance, in KP lung tumours (scale bar: 50 μm); *n* = 3.

Data information: In the box plots, the centre line reflects the median, the cross represents the mean, and the upper and lower box limits indicate the first and third quartiles. Whiskers extend 1.5× the IQR. *$P < 0.05$; **$P < 0.01$. Two-tailed *t*-test. See also Appendix Table S3 (exact *P*-values and statistical test used).

© 2020 The Authors

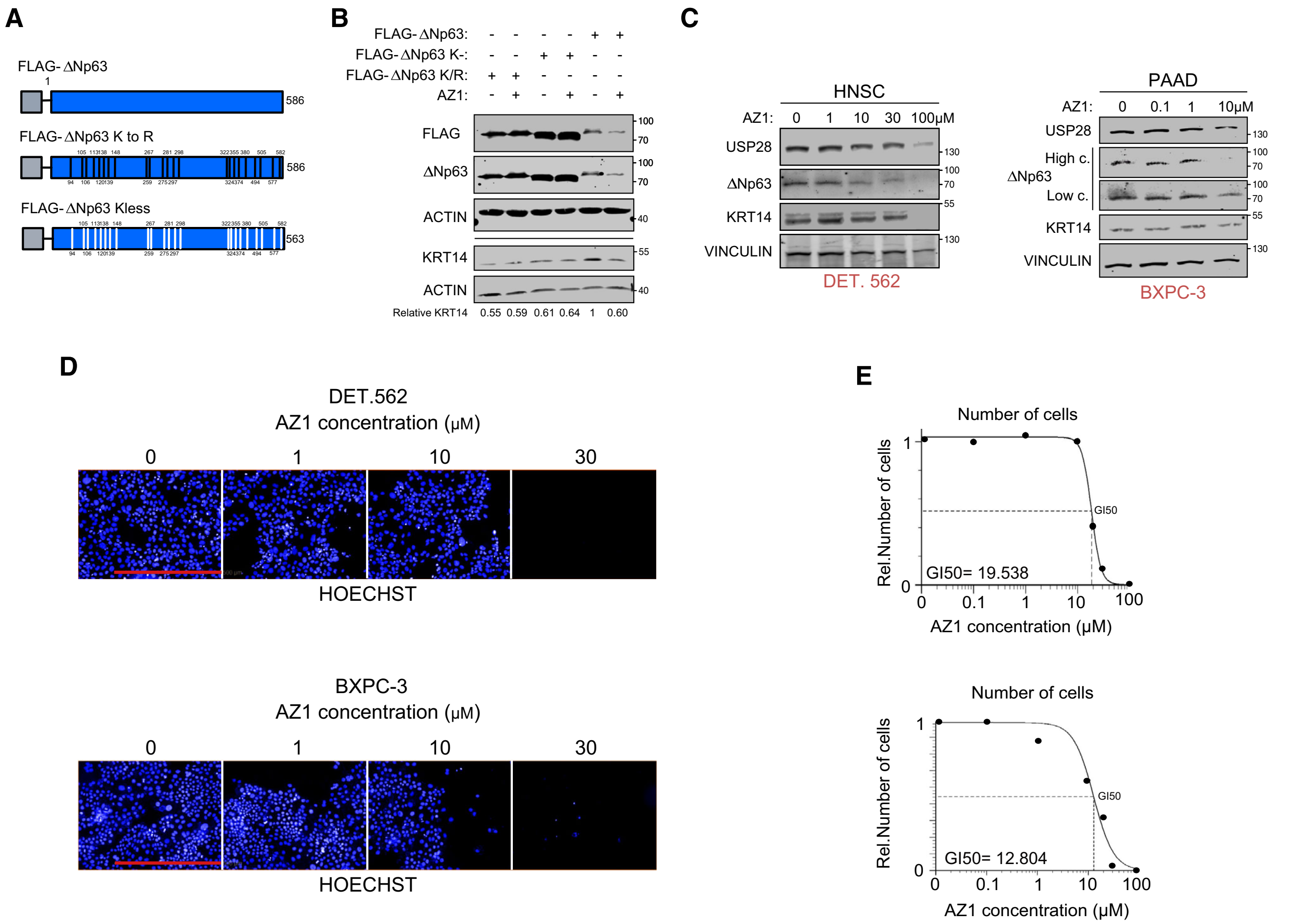

GI50 for AZ1

Primary Tumour:	Cell Line	ΔNp63	GI50(μM)
Vulva SCC	A-431	+	18.86
Lung SCC	LUDLU-1[adh]	+	1.55
Lung ADC	H1299	-	21.80
Cervix SCC	Ca Ski	+	14.61
Cervix SCC*	SiHa*	-*	30.02
Cervix ADC	Hela	-	21.82
HNSC SCC	Det. 562	+	19.53
PAAD SCC	BXPC-3	+	12.80

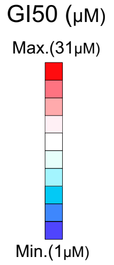

Figure EV4. Pharmacologic inhibition of USP28 with AZ1 regulates ΔNp63 stability via deubiquitylation and shows a selective anti-proliferative response of SCC cells.

A Schematic model of FLAG- ΔNP63, FLAG-ΔNP63 KtoR and ΔNP63 Kless mutant constructs.

B Immunoblot of FLAG, ΔNp63 and KRT14 in control and AZ1 (15 μM for 48 h)-treated, transiently transfected, HEK293T cells overexpressing FLAG-ΔNP63, FLAG-ΔNP63 KtoR or FLAG-ΔNP63 Kless. ACTIN as a loading control; $n = 3$; relative KRT14 was calculated using ACTIN as a loading control.

C Immunoblot of endogenous USP28, ΔNp63 and KRT14 in DET.562 and BXPC-3 cells treated for 24 h with either DMSO or indicated concentrations of AZ1. VINCULIN served as loading control; $n = 3$.

D DET.562 and BXPC-3 cells were seeded at equal cell density and cultured in the presence of either DMSO, 1, 10 or 30 μM AZ1 for 48 h. Scale bar = 500 μm.

E Cells were seeded at equal cell density and cultured in the presence of either DMSO, 0.1, 1, 10, 20 or 30 μM AZ1 for 48 h. Number of cells was quantified with the Operetta imaging system using Hoechst staining. 50% growth inhibition (GI50) was calculated. $n = 30$ fields analysed from independent wells.

F Table summarizing primary tumour, ΔNp63 status and GI50 for AZ1 of the different cancer cell lines analysed, red labelling in primary tumour = SCC; blue labelling in primary tumour = ADC; intense red box in GI50 = high-concentration AZ1. Intense blue box in GI50 = low-concentration AZ1. SiHa* = notably, the human Cervix SCC cell line SiHa was negative for ΔNp63.

Source data are available online for this figure.

 © 2020 The Authors

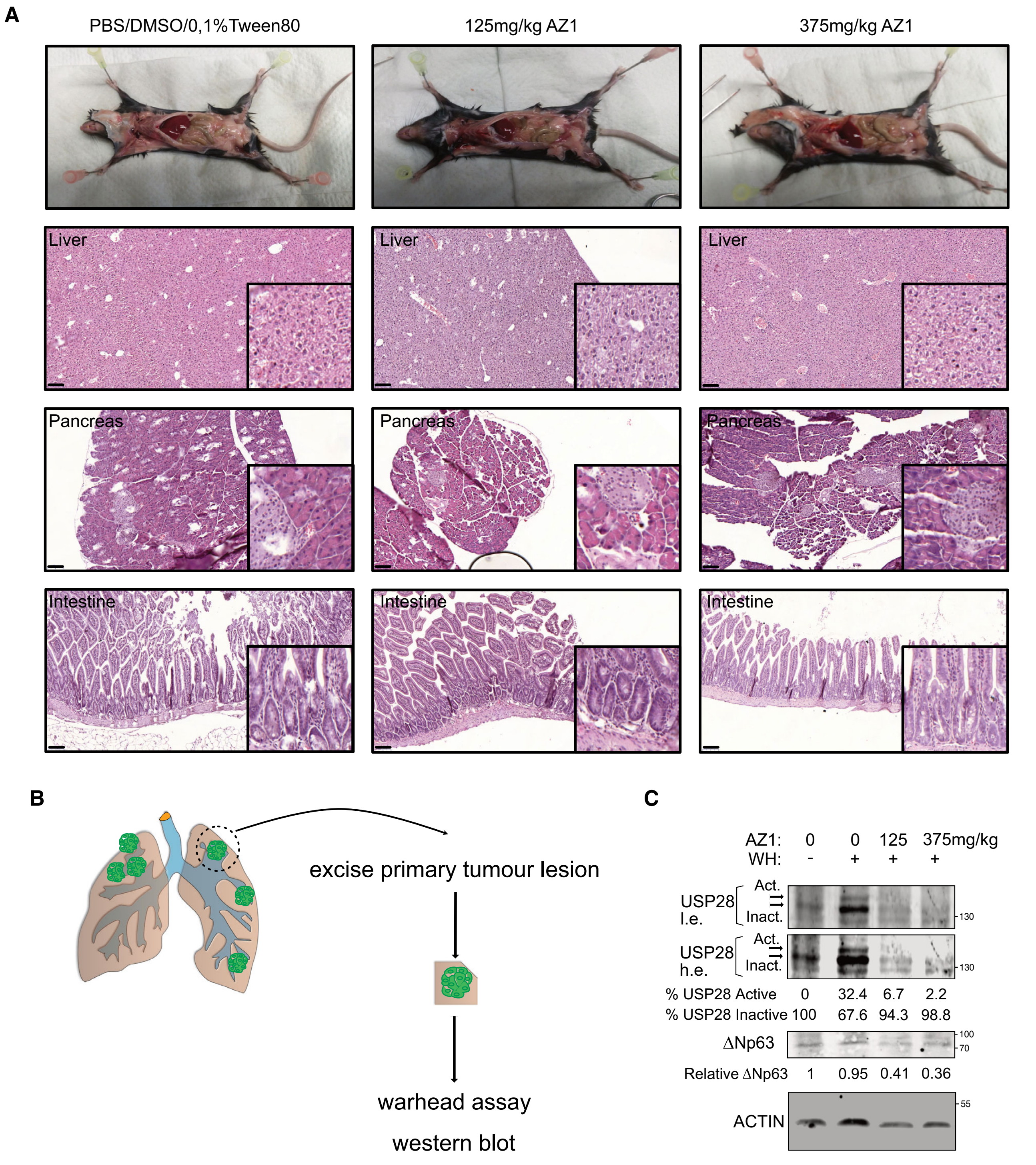

Figure EV5. Pharmacologic inhibition of USP28 with AZ1 reduces tumour growth in an orthotopic model of lung SCC tumours.

A Macroscopic and histological (representative H&E stainings from liver, pancreas and intestine) analysis of organs from animals treated with either control, solution, 125 or 375 mg/kg AZ1 (scale bars = 100 μm); *n* = 3.

B Schematic model for total tumour protein extraction from animals shown in (A).

C Representative ubiquitin-suicide probe immunoblot of endogenous USP28 from tumour explants as shown in (B). Shown are low (l.e.) and high exposure (h.e.) images of the same USP28 blot. Values indicate relative % of active or inactive USP28. Representative immunoblot of endogenous ΔNp63 from control or AZ1-treated animals. Values indicate relative expression of ΔNp63. ACTIN served as loading control; *n* = 3.

Source data are available online for this figure.

© 2020 The Authors
