# Supplementary figures and images for: Maintaining protein stability of ∆Np63 via USP28 is required by squamous cancer cells
Source: EMBO Mol Med. 2020 Mar 4;12(4):e11101. doi: 10.15252/emmm.201911101 (PMC7136964; doi:10.15252/emmm.201911101)

Fig.EV5C

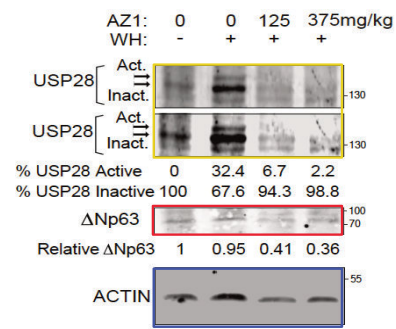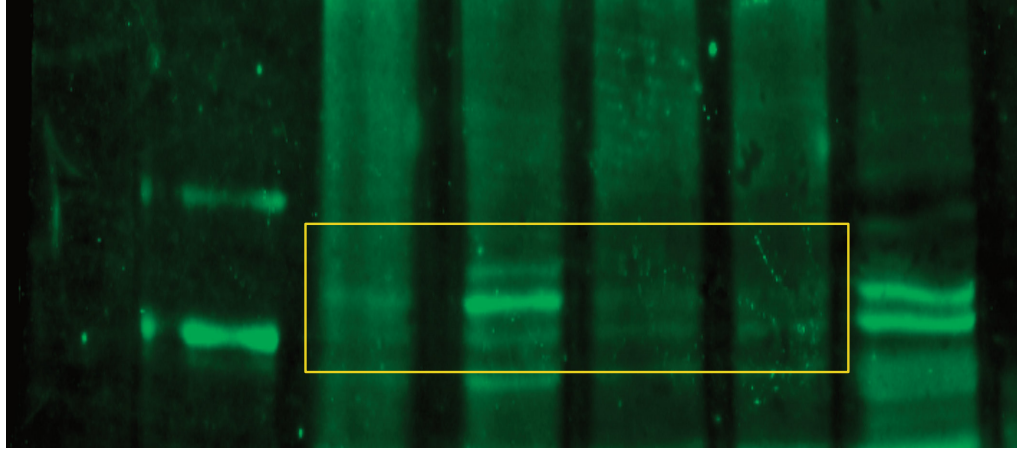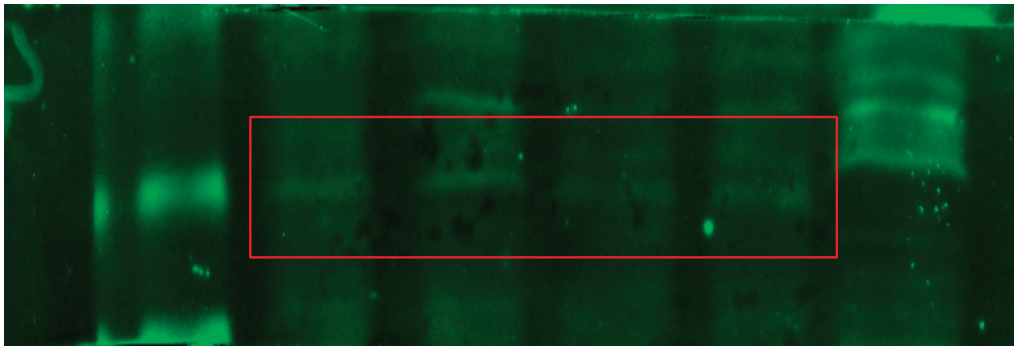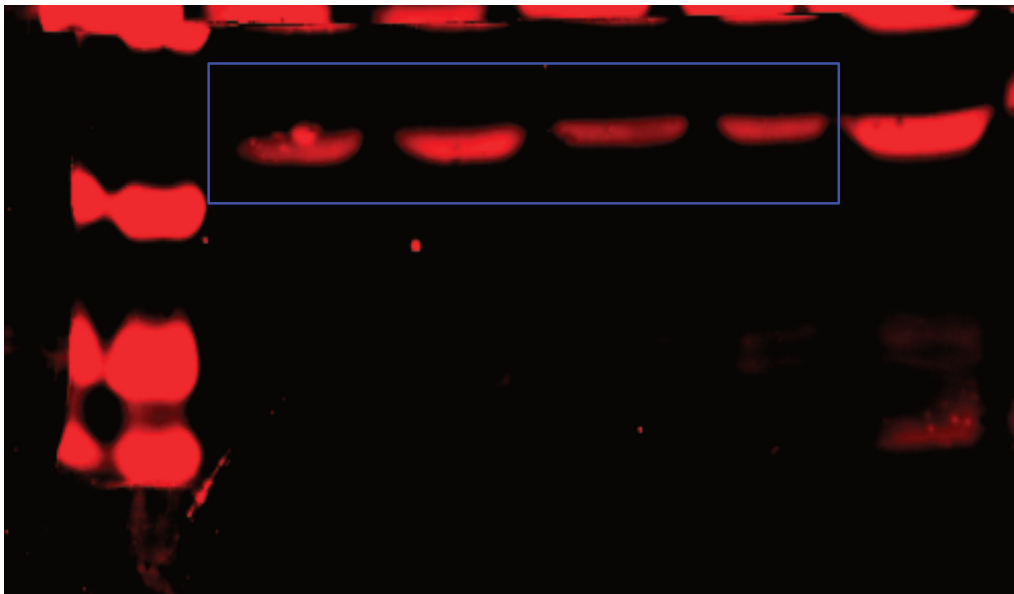

Supplement: Supplementary file 3 — Source Data for Expanded View [file EMMM-12-e11101-s007.zip › Fig.EV5_Unproc._western_blots.pdf]

Fig.3A

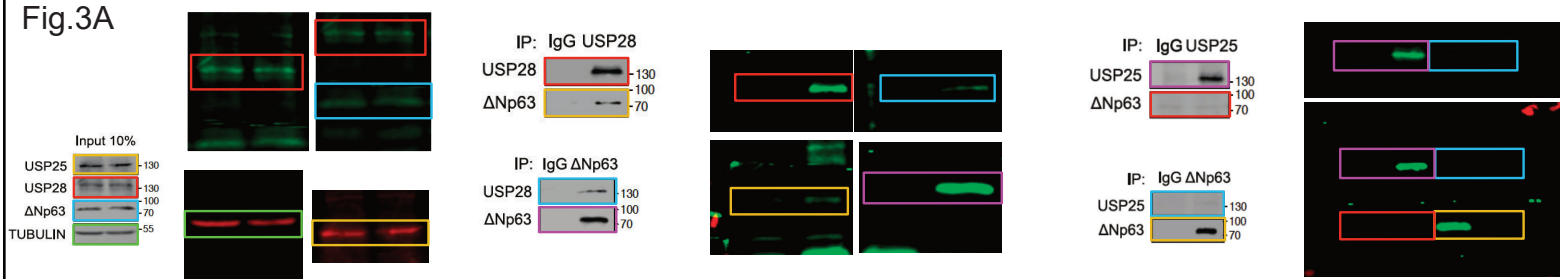

Fig.3B

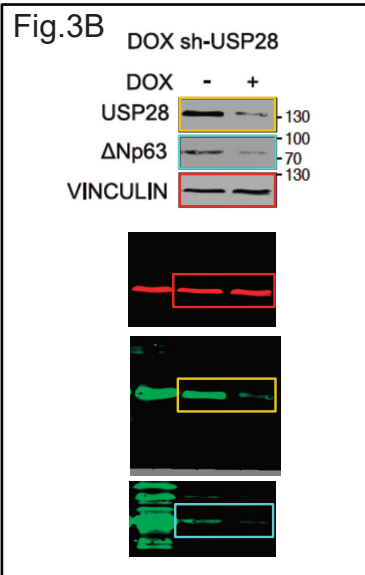

Fig.3C

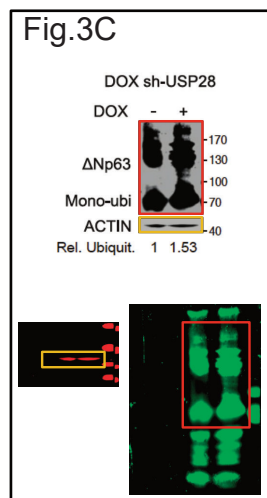

Fig.3D

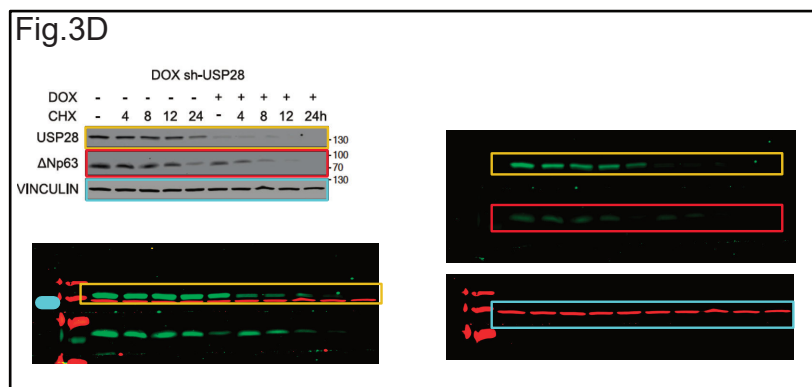

Fig.3E

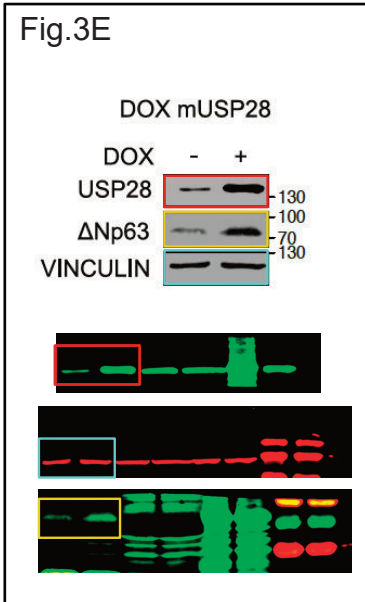

Fig.3F

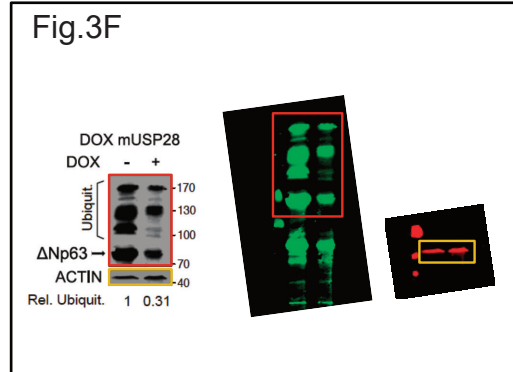

Fig.3H

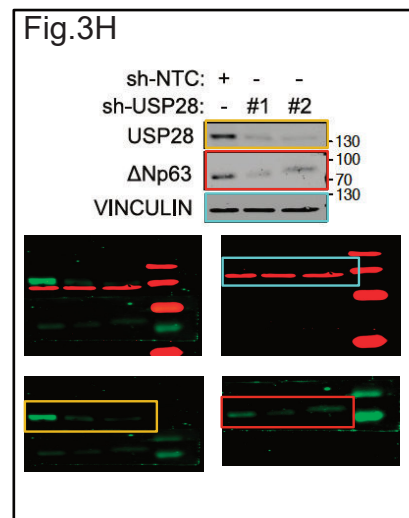

Fig.3G

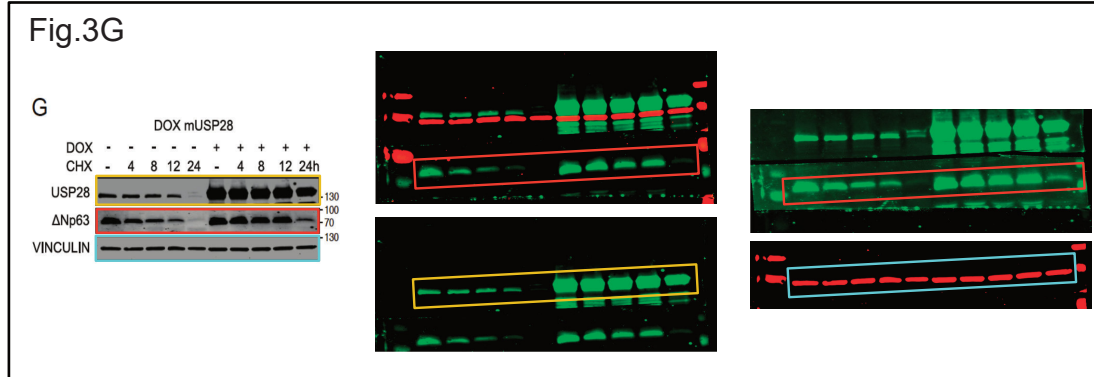

Fig.3I

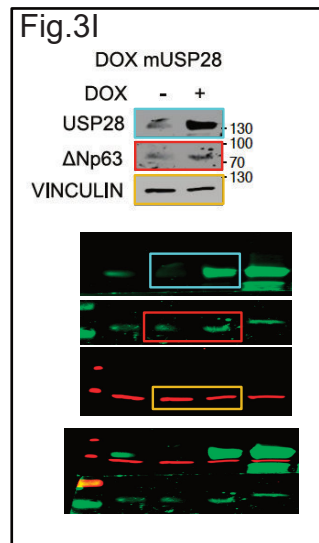

Supplement: Supplementary file 5 — Source Data for Figure 3 [file EMMM-12-e11101-s003.pdf]

# ACTIN line2 FIG6E NSCLC

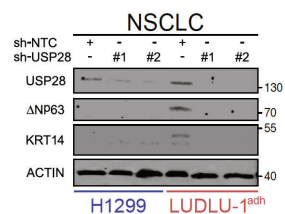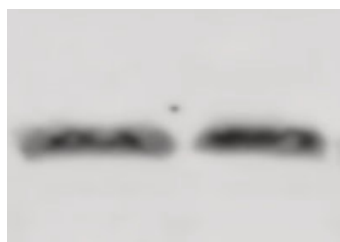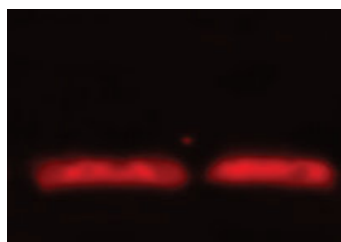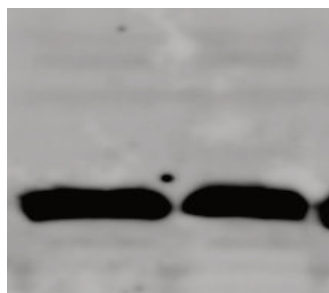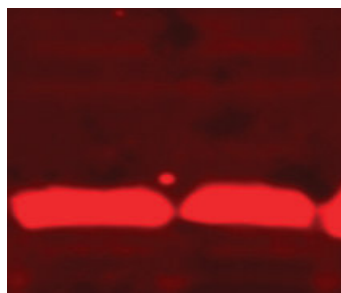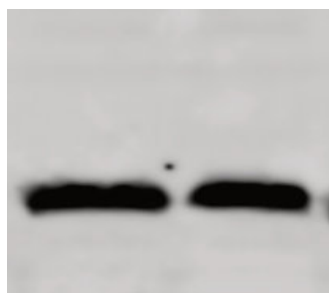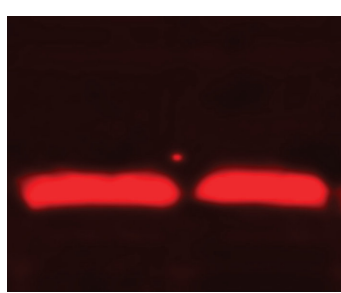

# ACTIN line2 FIG6E CERVIX

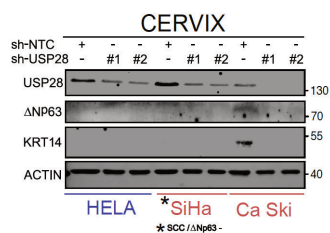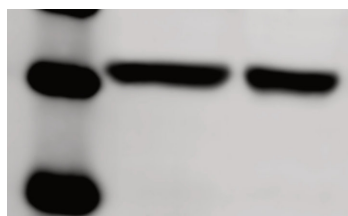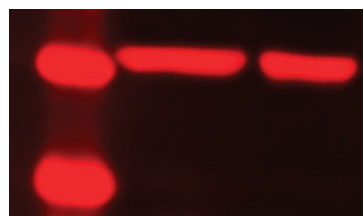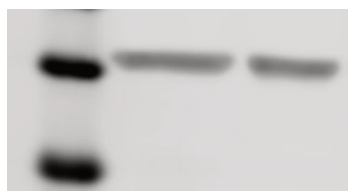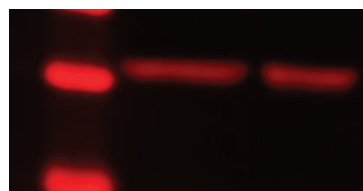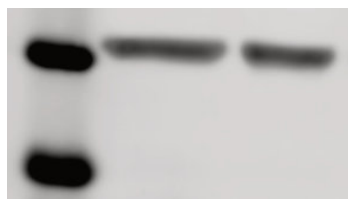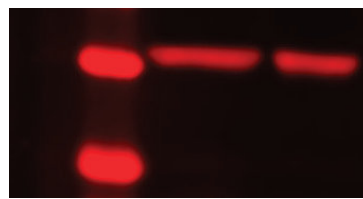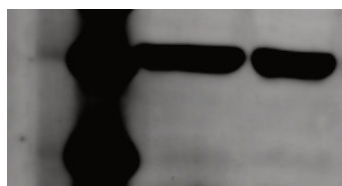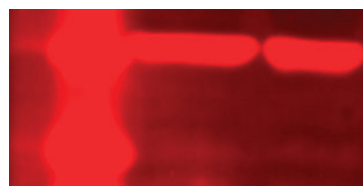

Supplement: Supplementary file 6 — Source Data for Figure 5 [file EMMM-12-e11101-s004.pdf]

ACTIN line2  
FIG6E  
PAAD

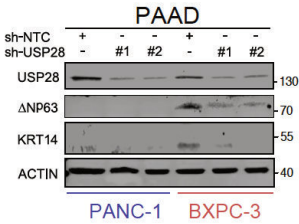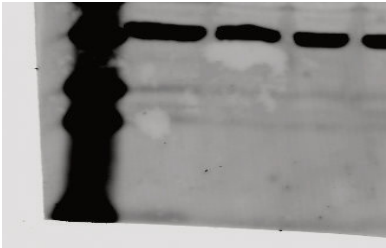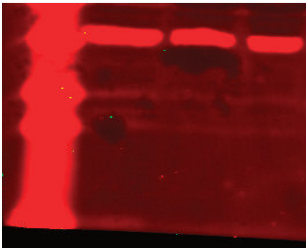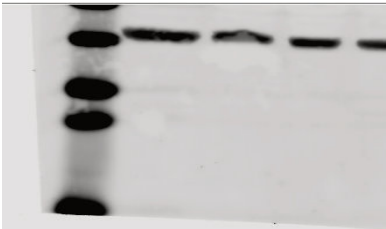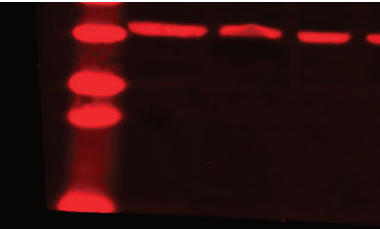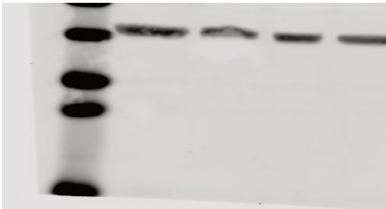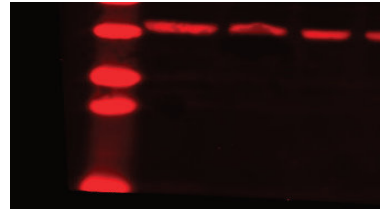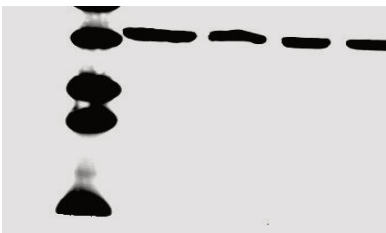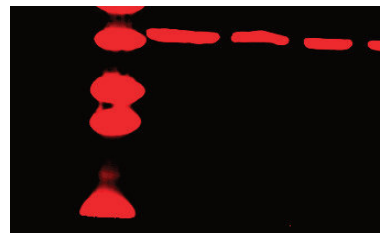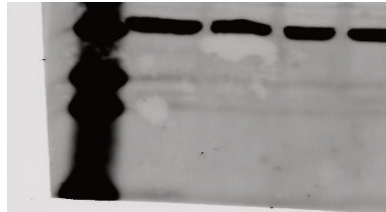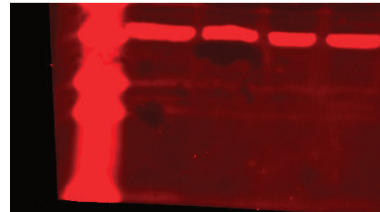

Supplement: Supplementary file 7 — Source Data for Figure 6 [file EMMM-12-e11101-s005.zip › 2line_actin_FIg6E_PAAD.pdf]

Fig.6E

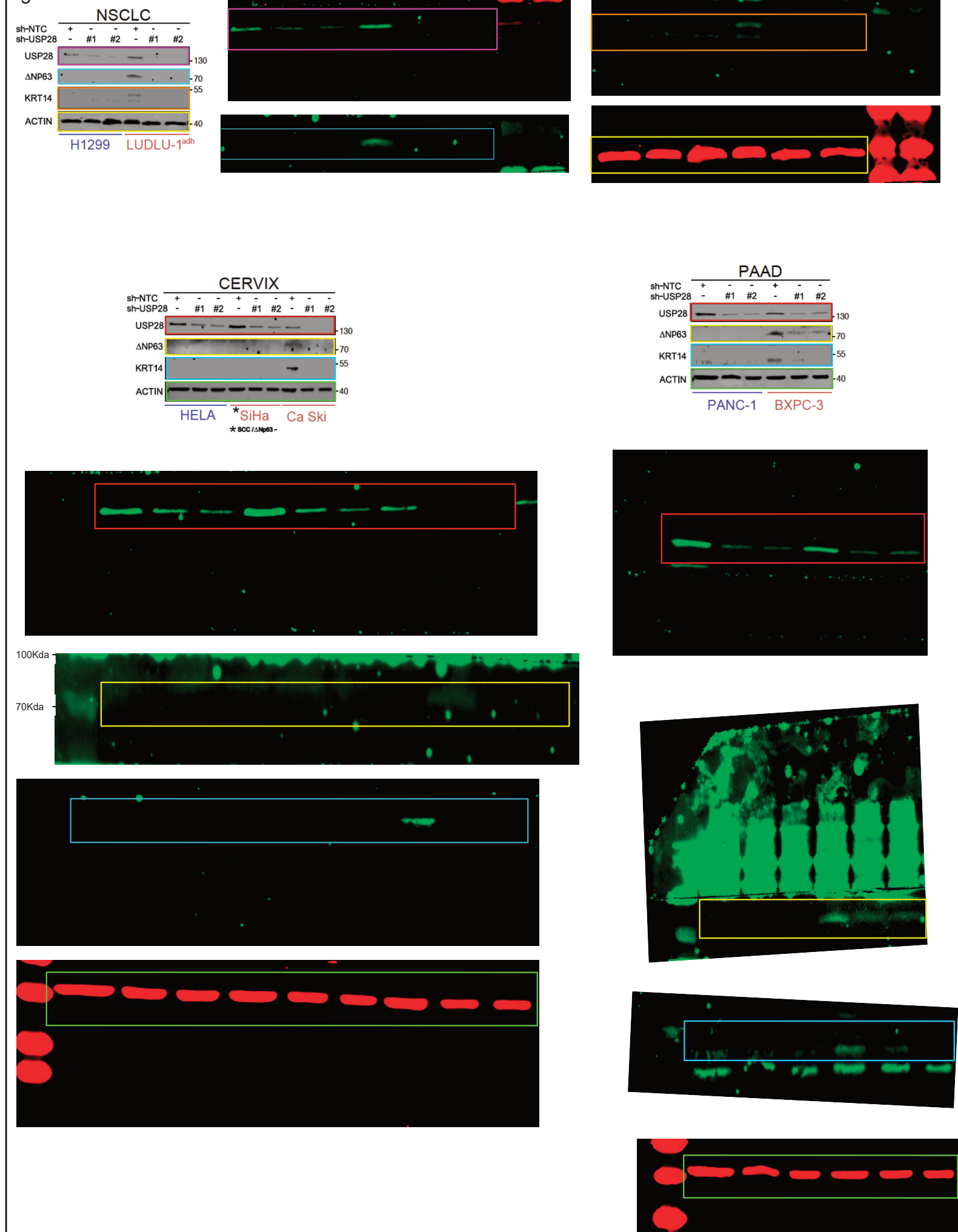

Supplement: Supplementary file 7 — Source Data for Figure 6 [file EMMM-12-e11101-s005.zip › Fig.6_Unproc._western_blots.pdf]

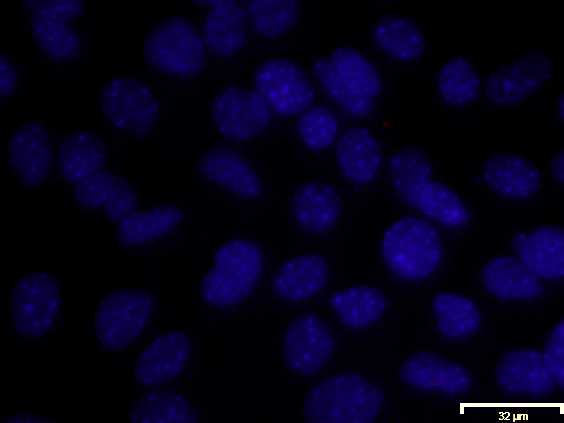

Supplement: Supplementary file 8 — Source Data for Figure 8 [file EMMM-12-e11101-s006.zip › FIG8 single images IF/kp1 p63 4a4_composite.Tif]

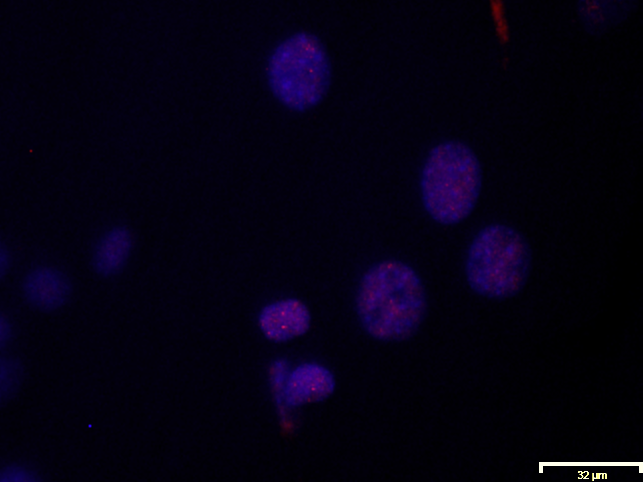

Supplement: Supplementary file 8 — Source Data for Figure 8 [file EMMM-12-e11101-s006.zip › FIG8 single images IF/kpl6-2 p63 4a4_composite.Tif]

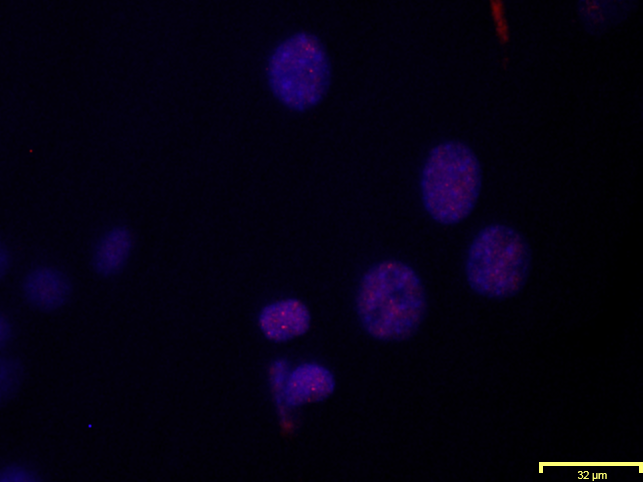

Supplement: Supplementary file 8 — Source Data for Figure 8 [file EMMM-12-e11101-s006.zip › FIG8 single images IF/kpl6-2 p63 4a4_merge.Tif]

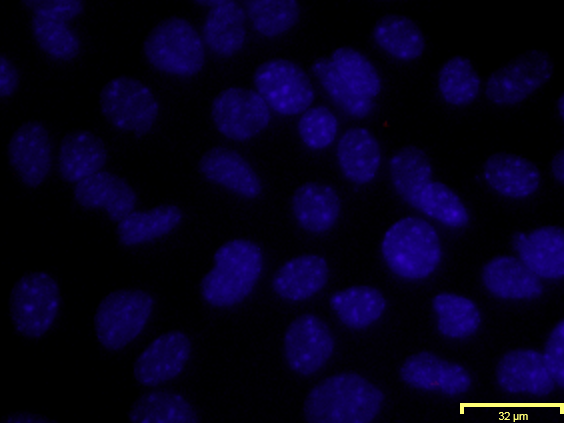

Supplement: Supplementary file 8 — Source Data for Figure 8 [file EMMM-12-e11101-s006.zip › FIG8 single images IF/kp1 p63 4a4_merge.Tif]

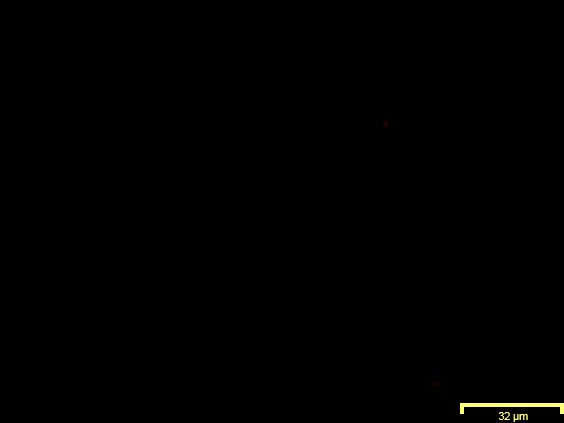

Supplement: Supplementary file 8 — Source Data for Figure 8 [file EMMM-12-e11101-s006.zip › FIG8 single images IF/kp1 p63 4a4_single channel Alexa 555.Tif]

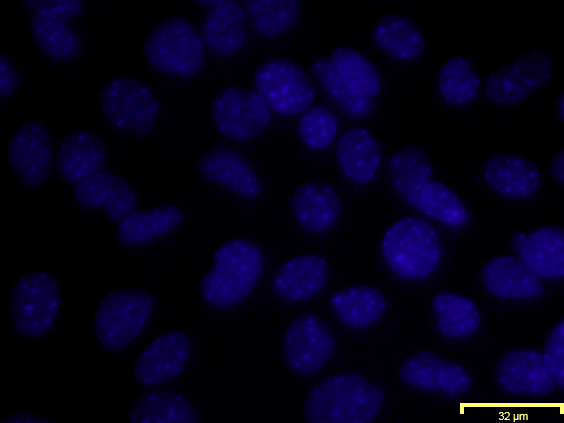

Supplement: Supplementary file 8 — Source Data for Figure 8 [file EMMM-12-e11101-s006.zip › FIG8 single images IF/kp1 p63 4a4_single channel dapi.Tif]

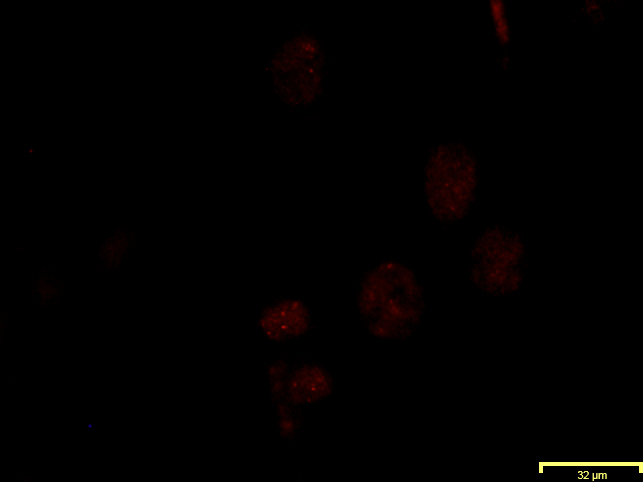

Supplement: Supplementary file 8 — Source Data for Figure 8 [file EMMM-12-e11101-s006.zip › FIG8 single images IF/kpl6-2 p63 4a4_single channel Alexa 555.Tif]

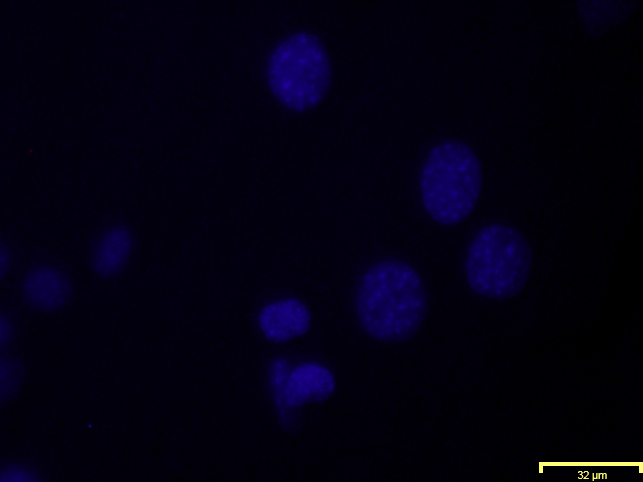

Supplement: Supplementary file 8 — Source Data for Figure 8 [file EMMM-12-e11101-s006.zip › FIG8 single images IF/kpl6-2 p63 4a4_single channel Dapi.Tif]
