# Supplementary material for: Maintaining protein stability of ∆Np63 via USP28 is required by squamous cancer cells
Source: EMBO Mol Med. 2020 Mar 4;12(4):e11101. doi: 10.15252/emmm.201911101 (PMC7136964; doi:10.15252/emmm.201911101)

Fig.EV4B

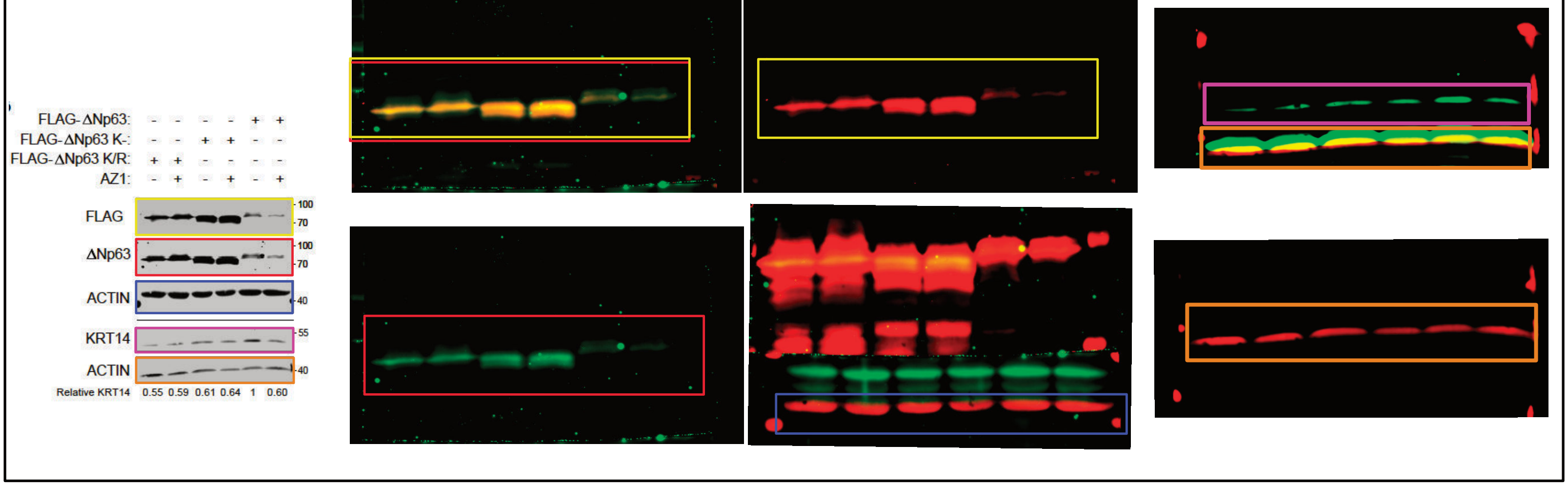

Fig.EV4B

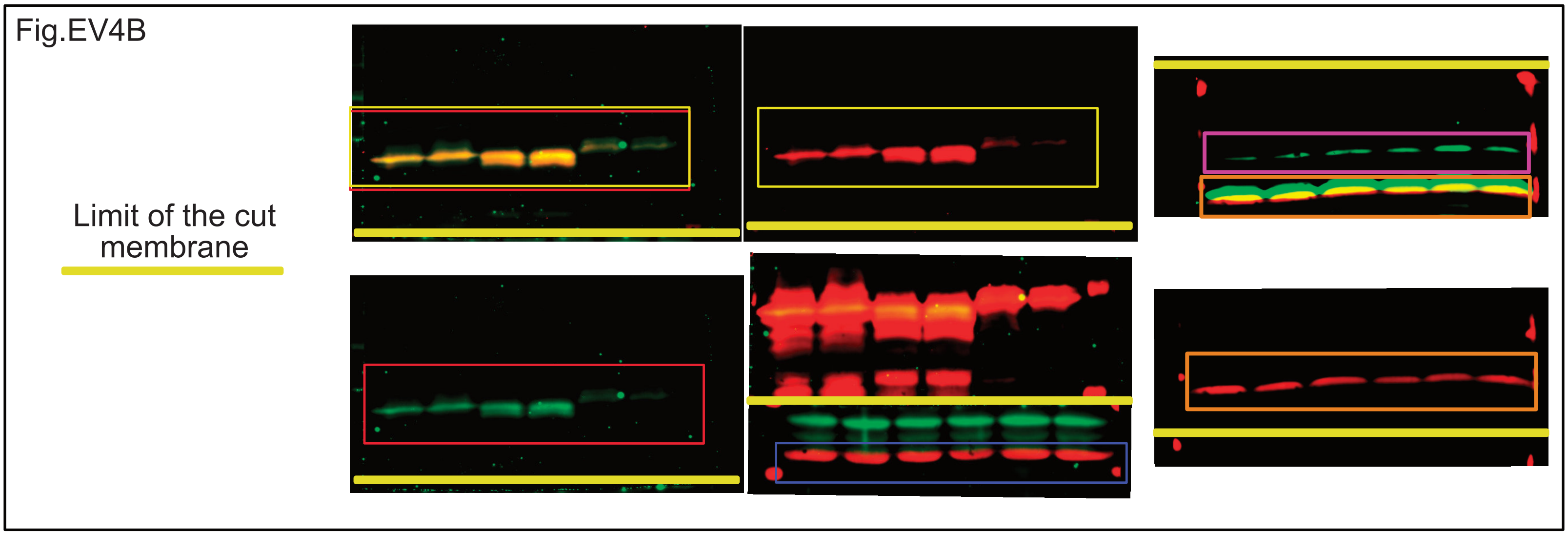

Supplement: Supplementary file 3 — Source Data for Expanded View [file EMMM-12-e11101-s007.zip › Fig EV4 Source Data.zip!/FIGEV4B_Unproc._western_blots_with_cut.pdf]
